# Case studies on potential G-quadruplex-forming sequences from the bacterial orders *Deinococcales* and *Thermales* derived from a survey of published genomes

**DOI:** 10.1038/s41598-018-33944-4

**Published:** 2018-10-24

**Authors:** Yun Ding, Aaron M. Fleming, Cynthia J. Burrows

**Affiliations:** Department of Chemistry, University of Utah, 315 South 1400 East, Salt Lake City, UT 84112-0850 United States

## Abstract

Genomes provide a platform for storage of chemical information that must be stable under the context in which an organism thrives. The 2‘-deoxyguanosine (G) nucleotide has the potential to provide additional chemical information beyond its Watson-Crick base-pairing capacity. Sequences with four or more runs of three G nucleotides each are potential G-quadruplex forming sequences (PQSs) that can adopt G-quadruplex folds. Herein, we analyzed sequenced genomes from the NCBI database to determine the PQS densities of the genome sequences. First, we found organisms with large genomes, including humans, alligators, and maize, have similar densities of PQSs (~300 PQSs/Mbp), and the genomes are significantly enriched in PQSs with more than four G tracks. Analysis of microorganism genomes found a greater diversity of PQS densities. In general, PQS densities positively tracked with the GC% of the genome. Exceptions to this observation were the genomes from thermophiles that had many more PQSs than expected by random chance. Analysis of the location of these PQSs in annotated genomes from the order *Thermales* showed these G-rich sequences to be randomly distributed; in contrast, in the order *Deinococcales* the PQSs were enriched and biased around transcription start sites of genes. Four representative PQSs, two each from the *Thermales* and *Deinococcales*, were studied by biophysical methods to establish the ability of them to fold to G-quadruplexes. The experiments found the two PQSs in the *Thermales* did not adopt G-quadruplex folds, while the two most common in the *Deinococcales* adopted stable parallel-stranded G-quadruplexes. The findings lead to a hypothesis that thermophilic organisms are enriched with PQSs as an unavoidable consequence to stabilize thermally their genomes to live at high temperature; in contrast, the genomes from stress-resistant bacteria found in the *Deinococcales* may utilize PQSs for gene regulatory purposes.

## Introduction

The central dogma of biology determines the flow of information from DNA to proteins. Regulation of the information flow occurs at many checkpoints, the first occurs at the level of the genome within promoters as well as in 5′- and 3′-untranslated regions (UTRs). Lastly, some genomes contain important sequences such as the repeats found in telomeres that are responsible for protecting the ends of chromosomes, and these sequences provide a molecular clock on the basis of their length^1^. These functional sequences contain information that can exist beyond the primary sequence of the DNA.

**Figure 1:**
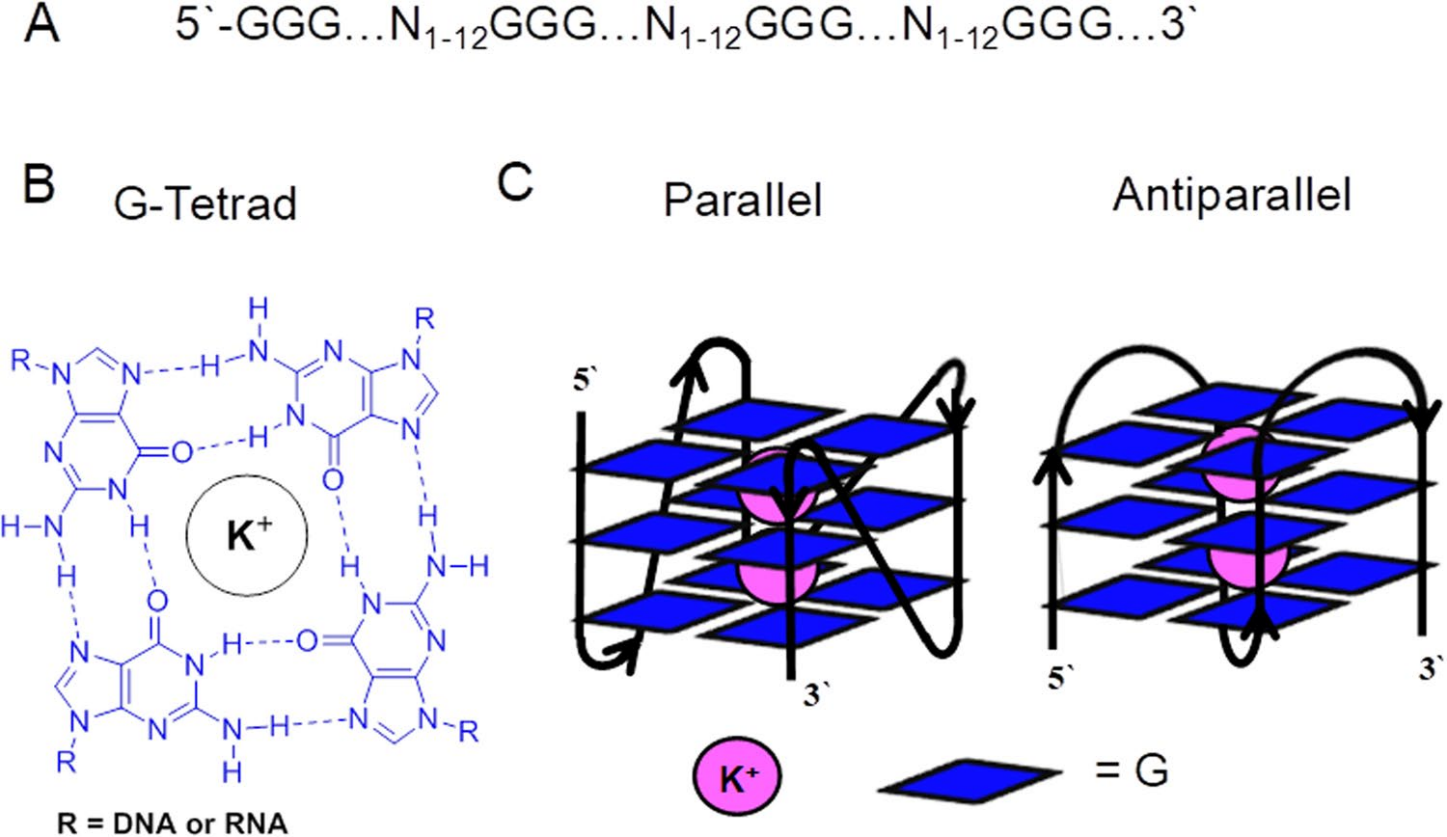
Characteristics of a G-quadruplex. (**A**) General sequence formula for a PQS. (**B**) Structure of a G-tetrad. (**C**) Cartoon representations for a parallel and antiparallel G4.

An additional layer of information found in a genome is the global and local structures the sequences can adopt. This Watson-Crick B helix is necessary for proteins, such as transcription factors, to scan DNA and locate their recognition sequences to regulate mRNA synthesis^2^. Additionally, local sequences may have the potential for adopting other secondary structures that include cruciform DNA^3^, Holliday junctions^4^, i-motifs in cytosine (C) rich regions^5,6^, and G-quadruplexes (G4) in guanine (G) rich regions of a genome^7–9^. Potential G-quadruplex forming sequences (PQSs) occur in DNA when four tracks of three or more Gs per track are located within 1–12 nucleotides between each track (Fig. 1A)^10^, although further interrogation of folded PQSs has found the loop lengths can be longer^11,12^. A PQS can adopt a G4 fold when one G from each of the four tracks come together to yield a G-tetrad held together by G:G Hoogsteen base pairs (Fig. 1B)^13–15^. Because three Gs exist in each run, three G-tetrads are formed and stack upon one another furnishing a structure with a central cavity in which intracellular K^+^ ions are coordinated. Structurally G4s are characterized as parallel or antiparallel stranded on the basis of the polarity of the four strands (Fig. 1C)^13–15^. In the genome, there exist many instances in which the PQSs possess more than four G tracks that can result in these sequences adopting dynamic structures equilibrating between multiple folds^16^. Cellular experiments regarding G4s and their impact on cellular activities have identified many functions for these non-B-form helical folds.

The ability of PQSs to fold intracellularly was first observed by immunostaining of ciliate telomeres^17^, and this study was followed by immunofluorescence of human cells to find folded G4s^18^. The folding of PQSs to G4s has been implicated in causing strand breaks during replication in the absence of faithful helicases to resolve these roadblocks to polymerase bypass^19^; when located in gene promoters, G4s can regulate transcription of the gene^20,21^; G4s may be important in telomere biology^15^; and they may function at origins of replication in humans^22^. Use of G4-ChIP-Seq identified ~10,000 folded G4s in human keratinocytes that up or down regulate expression of genes^21^. Experiments have found folded G4s are not limited to the genome as they can fold in the transcriptome^23,24^; however, this field of study remains a hot topic of debate in the current literature^25^.

The other fascinating property of G is its sensitivity toward oxidation because it has the highest electron density of the canonical bases^26,27^. Oxidative modification of the G nucleotide results in many downstream products, in which 8-oxo-7,8-dihydroguanine (OG) has garnered the most attention as a result of the levels of this oxidized heterocycle functioning as a bellwether of cellular oxidative stress^28^. We have found when G in the context of a promoter PQS is oxidized to OG, mRNA synthesis is impacted either up or down depending on the strand in which the PQS and OG resides (coding vs. template) in mammalian cells^29,30^. The change in gene expression was found to be initiated by coupling of the DNA repair process with transcriptional regulation. Similar observations of G oxidation in the context of promoter sequences capable of adopting a non-B form structure driving transcription have been documented by other laboratories^31,32^. Further, the DNA repair efficiency of oxidized G4s was found to be most efficient with more than four G tracks; the studies led to the proposal that having >4 G tracks allowed more flexibility of the G4 fold to accommodate the modified nucleotide facilitating the DNA repair process^16,33^. These reports have married the G-quadruplex and G oxidation fields and has set off a search for other organisms in which oxidation of G nucleotides in the PQS context may have regulatory features; however, as a first step to achieve this goal, inspecting genomes to locate PQSs of interest must occur.

The characteristic pattern that PQSs follow has enabled the development of algorithms to explore genome sequences for their identification^34–39^. This approach was first implemented on the human genome to identify >300,000 PQSs^34,35^, and deeper analysis of the data identified the PQSs were unequally distributed^40^. Direct sequencing of the human genome using G4-Seq located >700,000 folded sequences, in which the discrepancy with the bioinformatic value resulted from folded G4s with loops >12 nucleotides long and bulges between the Gs in adjacent G-tetrads^11^. The G-rich sequences were found to be biased toward gene promoters, 5′-UTRs, and the first intron, in addition to repeat sequences such as the telomere. This type of analysis now has been extended to select plant and microorganism genomes^41–47^, in addition to the DNA or RNA genomes found in viruses^48–53^. The massive implementation of more affordable next-generation DNA sequencing has enabled sequencing of thousands of genomes, particularly those from microorganisms.

In the present study, a bioinformatic analysis for PQSs in many sequenced higher organism genomes and all representative microorganism genomes found in the NCBI database was conducted. The findings were grouped and analyzed for trends across the classification hierarchy of the organisms. The gene annotations for these representative bacterial genomes such as those in the order of *Deinococcales* and *Thermales* allowed the determination of the PQS distributions in these organisms as a function of genomic element and the strand in which the G-rich sequences reside. The enrichments of PQSs in genes for biological processes and molecular functions were tested in some model organisms such as *Deinococcus radiodurans*. Finally, the two most common PQSs from the orders of *Deinococcales* and *Thermales* were characterized by standard biophysical methods to determine the G4 folding status of the sequences. The findings provide guidance for understanding PQSs in microorganism genomes and lay the foundation for a discussion regarding how PQSs have evolved to be distributed differently in these species.

## Materials and Methods

### Data retrieval

All representative complete genome files (.fna file) and genome annotation files (.gff3 file) were downloaded from the NCBI genome data base (download date = 08/10/2017) through the NCBI FTP services (1464 bacterial genomes and 184 archaea genomes). For the analysis of *Deinococcus*, *Thermus*, and *Cyanobacteria*, all possible complete genome files and genome annotation files were downloaded from the NCBI database. All other individual genomes and annotation files were obtained from the NCBI databases, which include Hg38 (human), CanFam3.1 (dog), MM9 (mouse), FelCat8 (cat), *Gallus Gallus* (chicken), *Oncorhynchus mykiss* (rainbow trout), *Chlamydomonas Reinhardtii* (v 3.1), *Methanosarcina acetivorans*, *Helicobacter pylori*, *Oryza sativa* (rice; v 4.0), *Arabidopsis thaliana* (assembly TAIR10.1), and Zm-PH207-REFERENCE_NS-UIUC_UMN-1.0 (maize). The meta data that include the GC% and the genome length were either obtained from the NCBI genome database (https://www.ncbi.nlm.nih.gov/genome/browse#!/prokaryotes/) or calculated by an in-house script. One noteworthy point is that the reference genomes for higher organisms do not contain sequences for telomere regions because their lengths are highly polymorphic; thus, the plots for organisms whose genomes have telomeres do not contain these PQSs.

### PQS density and distribution around the transcription start site

A modified Quadparser algorithm was used to search for PQSs in all genomes with allowed loop lengths between 1–12 nts and inspection for four or more G tracks with three or more Gs per track. The PQS density was defined as the number of PQSs per million base pairs (bps). The distributions of PQSs around transcription start sites (TSSs) were generated by an in-house Python 2.7 module (available at https://github.com/dychangfeng/6018_2017_final_project). Briefly, the TSSs of all genes were identified from the gff3 files and the distances between the PQSs and the closest TSSs were calculated from the Python module pybedtools (https://daler.github.io/pybedtools/). The distances were then binned (bin size = 30 bp) and exported to R 3.4.3 for further analysis and visualization. The analysis of PQS distribution in the coding versus template strands was achieved using the implemented functions in the Python scripts.

### Clustering and principal component analysis

Hierarchical clustering was performed in R 3.4.3 with the libraries “cluster” and “pvclust” (see the Github link for the script). First, the binned PQS distribution vector was normalized by the total number of PQSs for each bacterium. Then the “ward.D2” method was applied to calculate the distance matrix for all the bacteria belonging to the *Deinococcus* and *Thermus* orders. The dendrogram was achieved with the library “ggdendro” and bacteria from different orders of the *Deinococcus* and *Thermus* were color coded. Principal component analysis (PCA) was conducted from the “stats” library. Both scree plots and biplots were generated from the PCA results.

### Data visualization

All box plots, bar plots, dendrograms, and scatterplots were generated in R 3.4.3 with the library “ggplot2”.

### Gene ontology analysis

Gene lists around TSSs for some representative species (e.g., *Deinococcus radiodurans*) were produced from the in-house python module (https://github.com/dychangfeng/PQS_bacterial). The gene lists were then tested for enrichment of molecular pathways and biological process using the Gene Ontology database (http://www.geneontology.org/page/go-enrichment-analysis). The cutoff FDR was set at 0.05.

### Random genome simulation

Random genomes of 2 million bps were created at a specified GC% by a python script (see Github link). There were 10 random genomes generated at each GC% from 20% GC to 80% GC with 2% step size. The number of PQSs were identified by the regular expression of (5′-(G_≥3_N_1–12_)_≥3_G_≥3_-3′) in both strands. The algorithm employed inspected for PQSs with ≥4 G tracks that allowed determination of the number of PQSs with extra G tracks. Box plots of PQS density were generated at each GC% to form a theoretical line of PQS density vs. GC%.

### Biophysical Characterization of the G4 folds

Analysis to establish whether four selected PQSs could adopt G4 folds was achieved following previously outlined methods (see SI for complete details)^36,54^. After synthesis and purification of the PQSs, they were evaluated by ^1^H-NMR, circular dichroism (CD), and thermal melting analysis (*T*_*m*_), in which complete details to conduct these methods are reported in the Supporting Information.

## Results and Discussion

**Figure 2:**
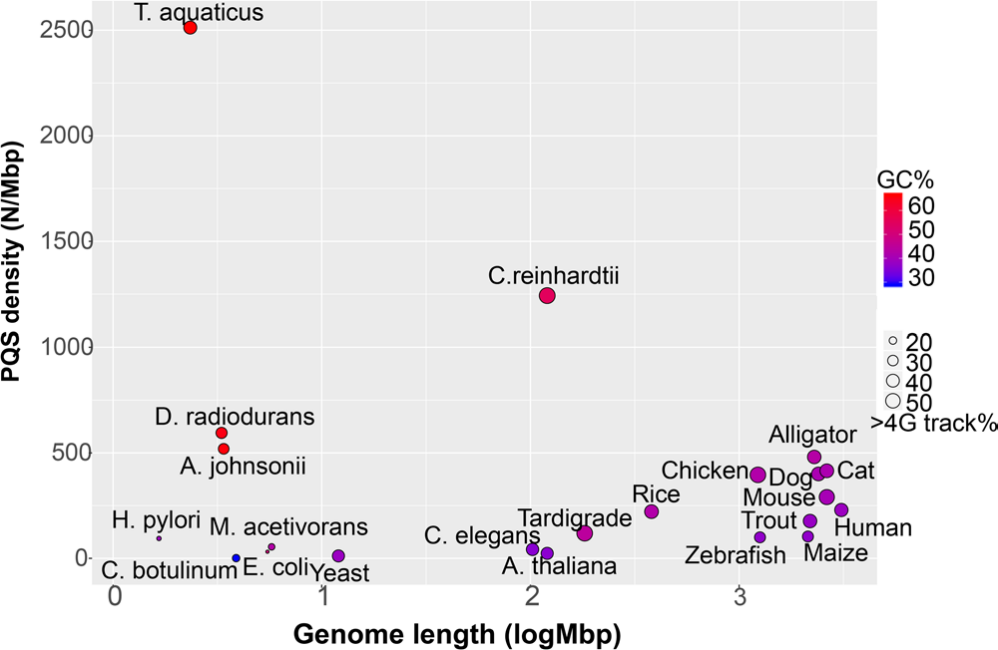
The density of PQSs in the genomes of select organisms as a function of the genome length, GC%, and percentage of PQSs with >4 G tracks.

Application of the algorithm to inspect for PQSs in genomes from a survey of prokaryotic and eukaryotic organisms was conducted. The data were plotted as the density of PQSs (i.e., number of PQSs/Mbp) with comparisons made to the genome length, GC%, and the percentage of PQS populations with >4 G tracks (Fig. 2). First, the plot identified a large distribution of PQS densities for the organisms surveyed. The organisms with large genomes (>10^9^ bps; log(Mbp) >3) all have similar GC% (36–44%) and possess PQSs with densities ranging from 100–500 per Mbp (low = maize and high = alligator). The data analyzed found 30–50% of the PQSs identified existed with more than four G tracks. The mouse and chicken genomes contain slightly more PQSs with >4 G tracks (50%). The human genome has a PQS density of 230 per Mbp that was in the middle of the PQS density range for the organisms evaluated with genomes >10^9^ bps. This initial finding suggests organisms with long genomes having similar GC% possess PQSs at nearly the same frequency.

In the large genome cohort, the percentage of PQSs with four or more G tracks was evaluated, and ~45% of the PQSs were found to have more than four G tracks, with the exception of the zebrafish and maize (Fig. 2). To address whether this is a significant enrichment in PQSs with more than four G runs, a series of randomized genomes with defined GC% from 20–70% with a length of 2 Mbps was created. Analysis of the randomized genomes with the PQS searching algorithm found that when the GC% = 40, the amount of PQSs with >4 G tracks represented ~10% of the population (Fig. S1); therefore, the finding that ~45% of the PQSs in large genome organisms having additional G runs beyond those necessary for G4 folding is significant. This observation suggests additional G tracks were favorably selected during evolution. Further, this supports our previous studies that identified these additional G tracks (i.e., “spare tires”) may function to maintain the G4 folding in the event of damage to the principle G4 structure^16^.

The initial analysis of the PQS content in organisms with smaller genomes found considerably more diversity than was detected in the larger genomes (Fig. 2). Major differences include greater variability in GC% ranging from 30–60%, the PQS densities ranged from nearly 0–2500 PQSs per Mbp, and the percentage of PQSs with >4 G tracks ranged from 20–50%. A few noteworthy observations are that many organisms have genomes with low numbers of PQSs that include the model organisms *E*. *coli*, yeast, *C*. *elegans*, and *A*. *thaliana*, as well as the anaerobic bacterium *C*. *botulinum*, the methane-producing bacterium *M*. *acetivorans*, and the gut bacterium *H*. *pylori*. The low PQS frequency in *E*. *coli*, yeast, and *A*. *thaliana* were previously reported^41,46^. The genomes of tardigrade (a.k.a., water bear) and rice have similar PQS densities as found in the large genome cluster (300 PQSs/Mbp). Organisms that had >500 PQSs/Mbp were *A*. *johnsonii* (519 PQSs/Mbp; 975 in the genome) found in the human microbiome, the radiation resistant bacteria *D*. *radiodurans* (595 PQSs/Mbp; 1,951 in the genome), the thermophile *T*. *aquaticus* (2,513 PQSs/Mbp; 5,277 in the genome), and the green algae *C*. *reinhardtii* (1,243 PQSs/Mbp; 149,160 in the genome). The organisms that had the highest PQS densities also possessed the greatest GC% in their genomes. We will discuss this point in more detail next. From the plot in Fig. 2, the greatest diversity in PQS densities was observed in the microorganism genomes that have not been fully analyzed for PQSs to date, and therefore, we analyzed more microorganism genomes from the NCBI database for PQSs.

**Figure 3:**
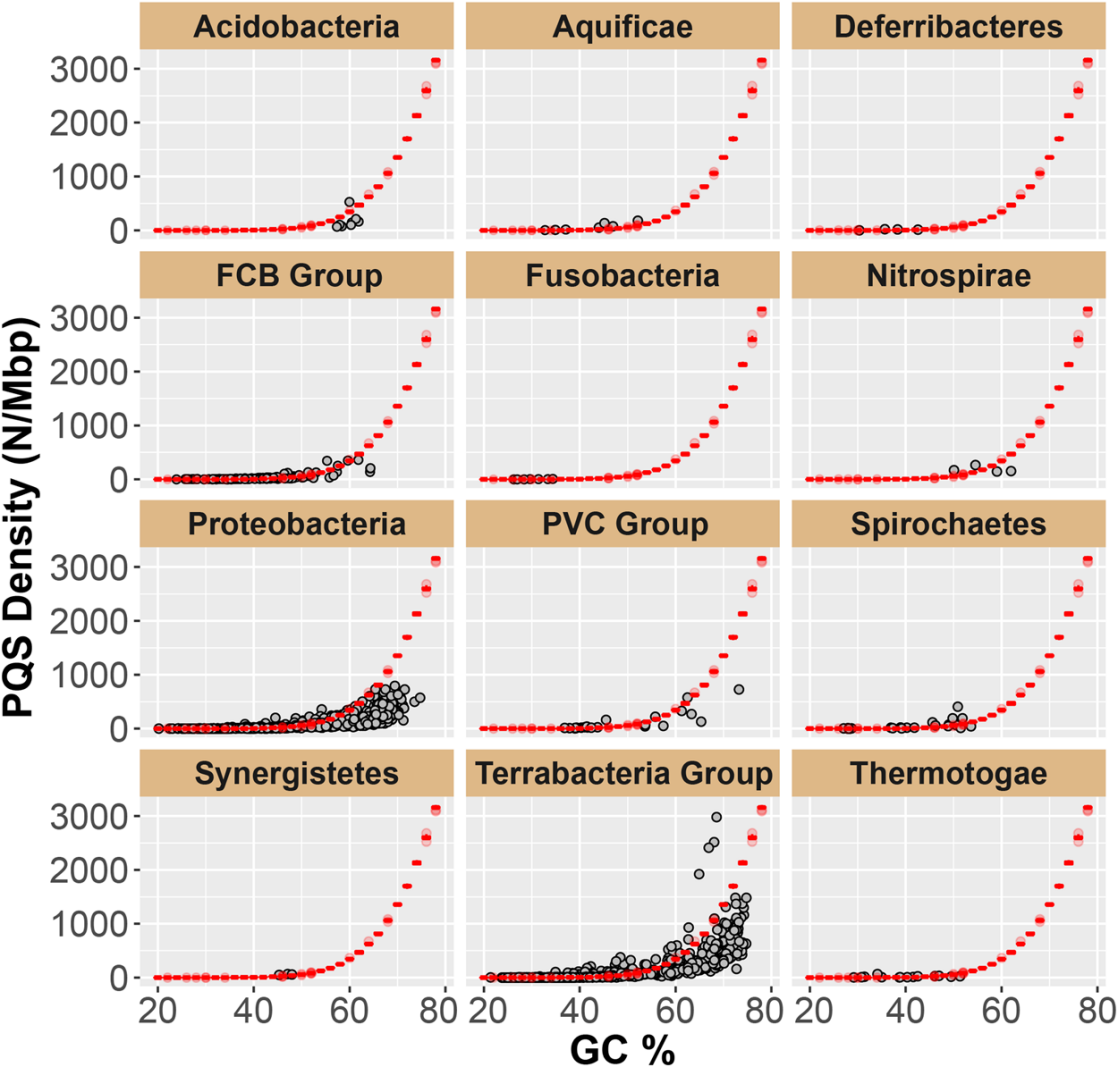
Plots of PQS densities and GC% for the phyla of bacteria with representative genomes found in the NCBI database. There exist six phyla of bacteria (*Caldiserica*, *Calditrichaeota*, *Chrysiogenetes*, *Dictyoglomi*, *Elusimicrobia*, and *Thermodesulfobacteria*) that have fewer than four representative genomes sequenced; these data can be found in the supporting information (Fig. S2). Three panels represent superphyla of bacteria that include the FCB group (phyla = *Fibrobacteres*, *Chlorobi*, and *Bacteroidetes*), PVC group (phyla = *Planctomycetes*, *Verrucomicrobia*, *Chlamydiae*, and *Lentisphaerae*), and the Terrabacteria group (phyla = *Cyanobacteria*, *Chlorflexi*, and *Deinococcus-Thermus*).

Analysis of each phylum of bacteria represented in the NCBI database using the PQS searching algorithm provided the data illustrated in Fig. 3. For each of the 12 phyla represented, the genomes analyzed were plotted with respect to the PQS densities and GC% (Fig. 3, black dots). Plotted alongside these data is a red curve representing the theoretical PQS density as a function of GC% that was derived from analyzing simulated genomes with defined nucleotide compositions (Fig. 3, red box plots). Inspection of the data in comparison to GC% identified that most bacterial genomes have PQSs at a density expected by random chance, particularly those with low GC%. Exceptions include the *Proteobacteria* with >50% GC that have PQS densities below the theoretical line. Bacteria in this *Proteobacteria* phylum appear to have genomes that selected against PQSs, on the basis of this observation. Notable genera in the *Proteobacteria* phylum include a wide variety of pathogens such as *Escherichia*, *Salmonella*, *Vibrio*, *Helicobacter*, *Yersinia*, and *Legionellales*.

**Figure 4:**
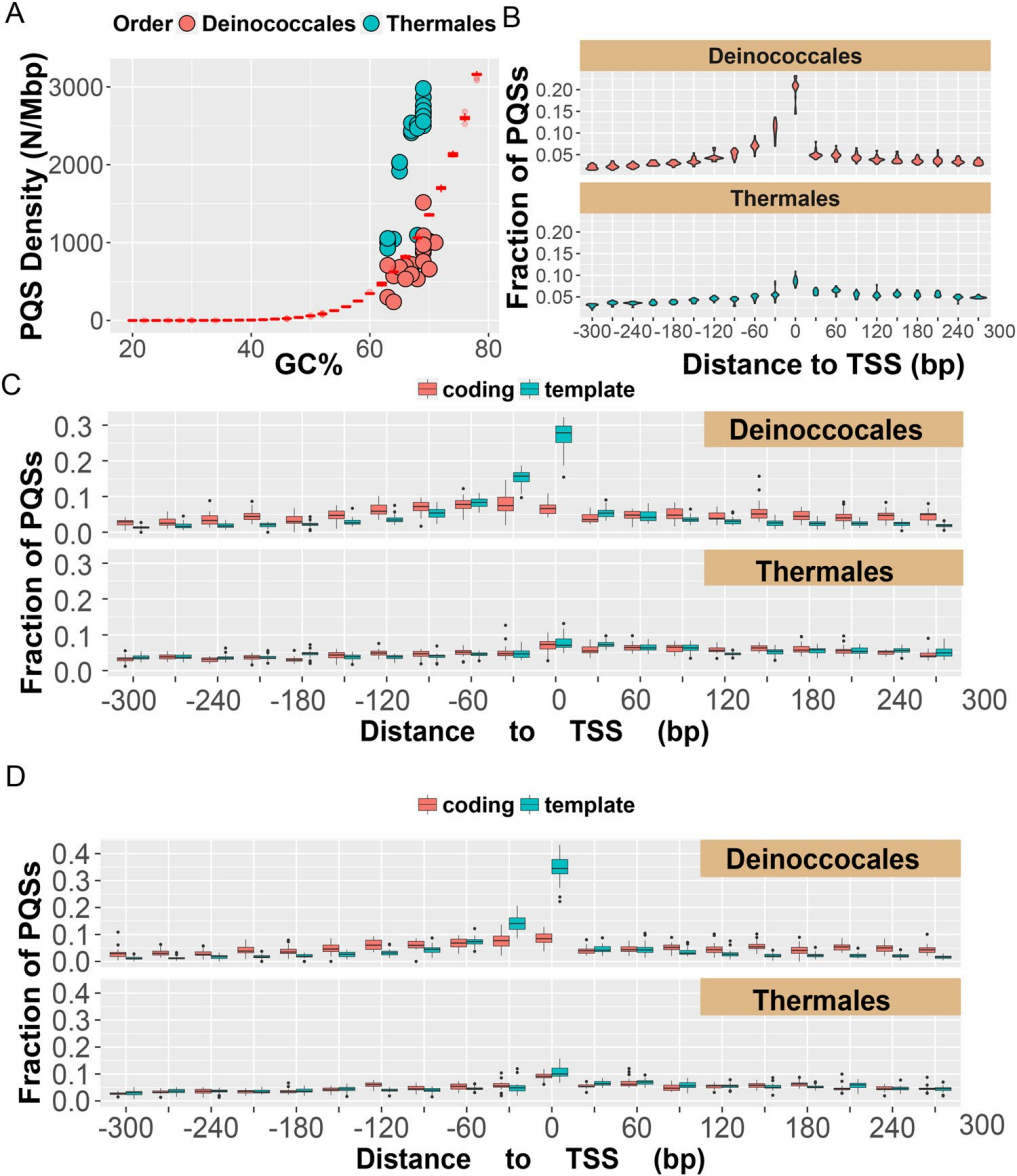
Profiles of the PQSs found in the phylum *Deinococcus-Thermus*. (**A**) Plot of densities of PQSs vs. GC%. The red box plots represent the theoretical PQS densities determined from analysis of randomized genomes with defined GC%. (**B**) Distribution of PQSs around TSSs binned at 30 nts. (**C**) Distributions of PQSs on the coding or template strand in the orders *Deinoccocales* and *Thermales*. (**D**) Distributions of >4 G-track PQSs on the coding or template strand in the orders *Deinoccocales* and *Thermales*.

The Terrabacteria group represents a superphylum of bacteria that includes the phyla *Cyanobacteria*, *Chlorflexi*, and *Deinococcus-Thermus* (Fig. 3); additionally, this superphylum contains nearly two-thirds of the known prokaryotes. In this group, a large number of the genomes analyzed had PQS densities close to the theoretical line or well below the line, with the exception of the *Deinococcus-Thermus* phylum. The bacteria in the *Deinococcus-Thermus* phylum have genomes with high GC% (>60%) and have high densities of PQSs (Fig. 4A). The *Deinococcus*- *Thermus* phylum represents two unusual orders of bacteria that are extremophiles. The order *Deinocccales* are known for their resistance to radiation, desiccation, toxic materials, ability to survive in the vacuum of space, as well as the capability to survive extreme heat and cold^55^. The order *Thermales* has bacteria that are resistant to heat, in which the notable member of this order is *T*. *aquaticus* that provided the thermo-stable DNA polymerase used in PCR. The *Deinococcus-Thermus* phylum has members whose genomes have been sequenced and annotated for genomic features (i.e., transcription start sites (TSSs), 5′- and 3′-UTRs, as well as coding regions) allowing an analysis of these genomes to determine whether the PQSs are distributed randomly or non-randomly throughout the genomes of these organisms.

Additional representative genomes from the NCBI database were inspected for PQSs. Quantification of PQS densities in the kingdom of Archaea found that the phyla *Methanomicrobia* and *Thermococci* to have greater densities of PQSs than the theoretical values based on the GC% of their genomes (Fig. S3). The *Thermococci* are extremeophiles found in hydrothermal vents, while *Methanomicrobia* represent a small group of Archaea that grow on methane (i.e., they are methanophiles) and can live at high temperatures. Next, analysis of the *Cyanobacteria* phylum for PQS density found that each representative genome contained about the same density of PQSs as predicted by the theoretical value based on the GC% of their genomes (Fig. S4). These additional analyses found greater densities of PQSs in thermophiles but no other organisms.

Following the previous observation that PQSs in the human genome are biased around key gene regulatory elements such as TSSs^40^, we inspected the *Deinococcus-Thermus* genomes with a focus for these G-rich sequences around the TSSs of annotated genes. First, the genomes analyzed were divided into the orders *Deinococcales* and *Thermales*, and then analyzed for PQS location relative to the TSS. Enrichment of PQSs around the TSSs was observed for the *Deinococcales* but not the *Thermales* (Fig. 4B). Next, the PQSs flanking the TSSs in these two orders were divided into those on the coding strand versus the template strand. In the *Deinococcales* order, the PQSs were found to be enriched on the template strand but not the coding strand^41,46^; in contrast, as expected, in the *Thermales* order showed no enrichment of PQSs on either strand (Fig. 4C). A similar dissection of PQSs around the TSSs of genes was conducted for archaea, and other orders found in the *Proteobacteria* phylum and the Terrabacteria group superphylum (Figs S5–S12). A few notable observations were made from this additional interrogation of the PQSs in these genomes. The *Xanthomonadales* order that comes from the *Proteobacteria* phylum and represents a collection of phytopathogens was found to possess PQSs highly enriched around the TSS. The orders *Pyrococcus* and *Thermococcus* found in the *Cyanobacteria* phylum were found to possess slightly enriched PQSs around the TSSs of many genes. The distribution of PQSs in *C*. *reinhardtii* is similar to that of the *Thermales* order, in which the PQS is evenly distributed throughout their genomes. These initial observations suggest PQSs might have different functions between those that have favored enrichment of these G rich sequences around regulatory regions of their genomes and those in which the PQSs are randomly distributed.

Inspired by our previous findings that PQSs with >4 G tracks are better equipped to accommodate oxidative modification of G nucleotides^16^, an analysis of the PQS distributions for those with >4 G tracks was commenced. The additional analysis identified greater enrichment of PQSs with >4 G runs around the TSSs of *Deinococcales* (p-value = 6.3 × 10^–6^) but not that of the *Thermales* (Fig. 4D). The finding of these additional G runs adds more support for the hypothesis that *Deinococcales* may utilize G4 folds under conditions of oxidative stress for gene regulation.

In the organisms that were found to have PQSs enriched around TSSs, the genes of enrichment were surveyed against the gene ontology (GO) database to determine whether any GO term is enriched with these PQS-containing genes in both molecular functions and biological pathways^56^. For example, *D*. *radiodurans*, which is a radiation-resistant bacterium, was found to have statistically significant enrichment of the GO term oxidoreductase activity in molecular functions when only genes with PQSs around the TSS on the template were considered (Table S1). Application of this analysis to another model organism *X*. *campestris* also found enrichment of the GO term oxidoreductase activity for the genes with PQSs around the TSS (−100 bp to 100 bp; Table S2). These observations hint that in some organisms, PQSs are enriched and biased around genes that code for proteins allowing these organisms to handle environmental stress; however, we note that this observation is not universal to all microorganisms.

**Figure 5:**
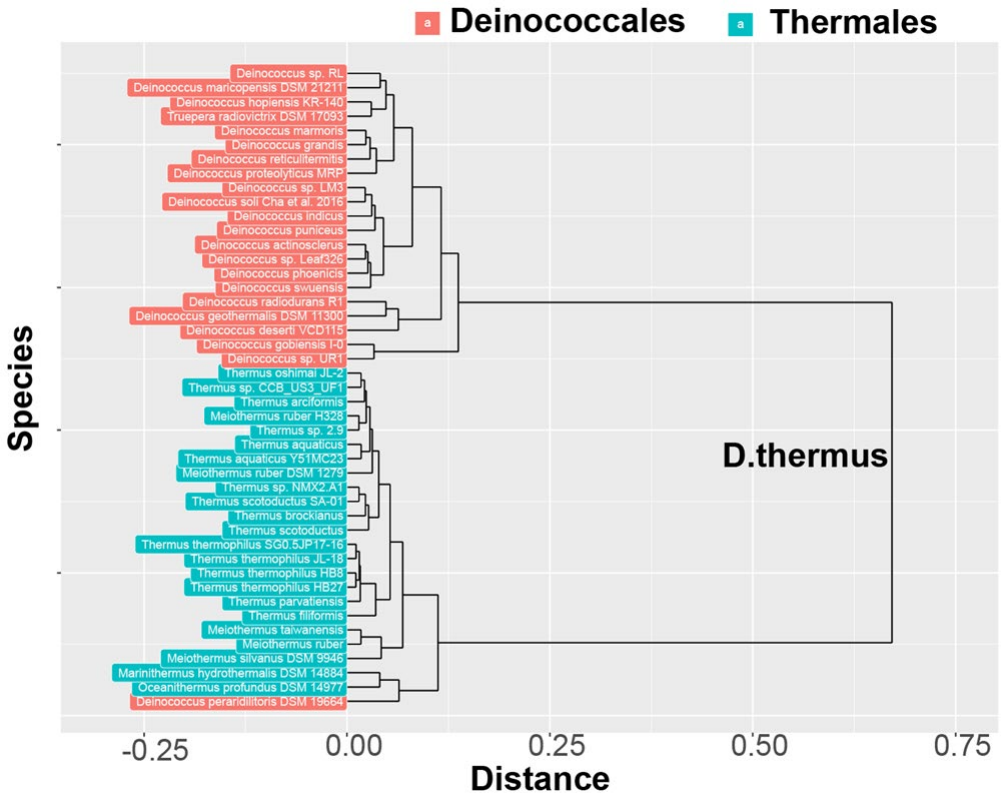
Hierarchical classification in the phylum *Deinococcus-Thermus* on the basis of the distribution of PQSs around TSSs. Unsupervised hierarchical classification was performed on the vectors of the PQS distributions around the TSSs. The bacterial names were colored coded after classification.

The distributions of PQSs enabled us to classify the bacteria on their PQS footprint on the genome. Principal component analysis (PCA) and hierarchical classification analysis were conducted on the PQS distributions around the TSSs for all the available genomes in the *Deinococcus-Thermus* phylum. The PCA analysis found two principal components (PC1 and PC2) during the unsupervised and unbiased reduction of the data to key components that accounted for 75% of the variance between all genomes in the cohort analyzed. Bacteria in the orders *Deinococcales* and *Thermales* were well separated in the dimensions of PC1 and PC2 (Fig. S13); this observation indicates the PQS distributions around the TSSs are characteristic to each order within the *Deinococcus-Thermus* phylum. The hierarchical classification of these PQS distributions showed that bacteria cluster into their own order with one exception (*D*. *peraridilitoris* DSM 19664). This fascinating observation from the PCA analysis of the distributions of PQSs is that the results agree with the hierarchical classification of each bacterial order (Fig. 5).

**Figure 6:**
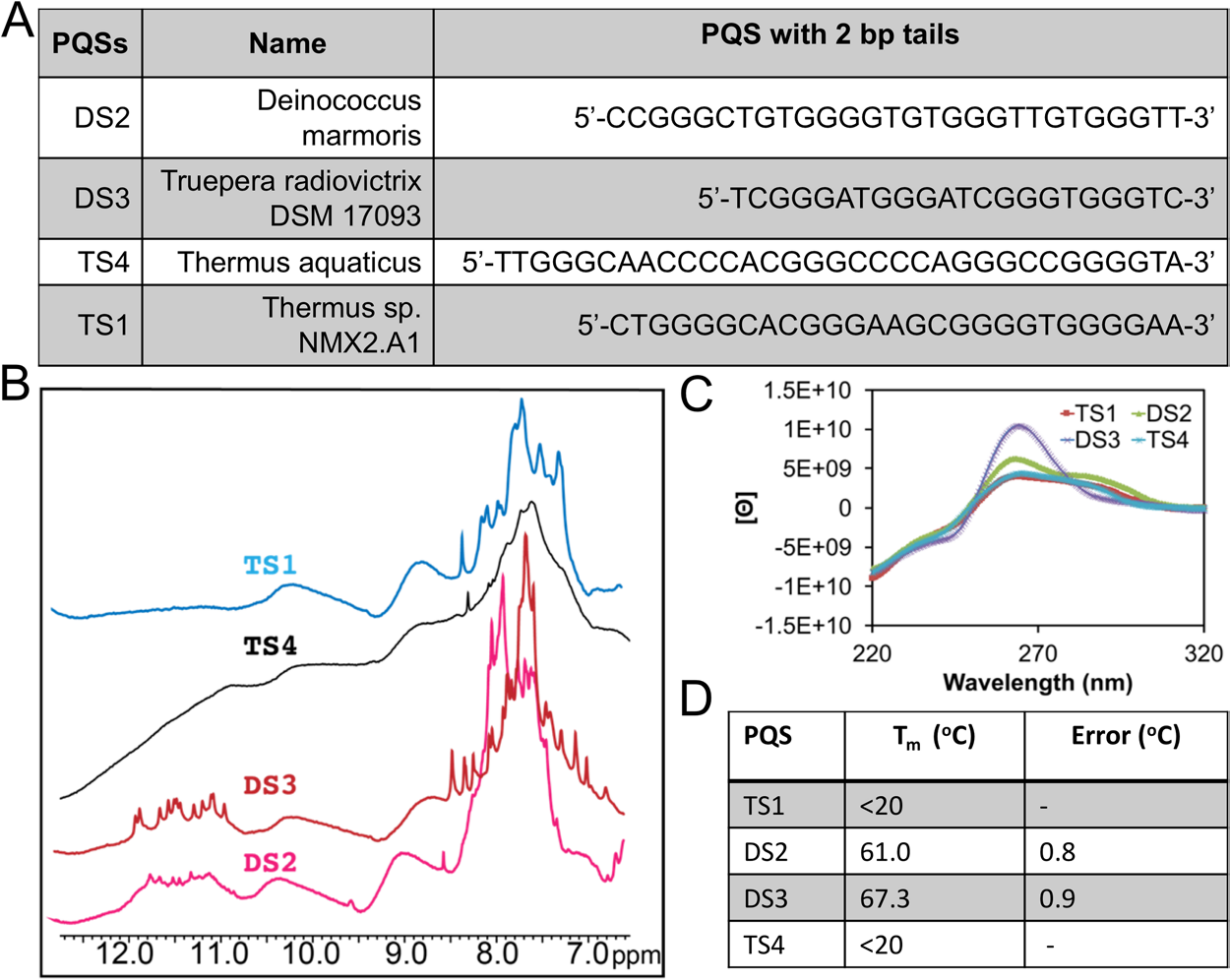
Representative PQSs selected for biophysical characterization to establish G4 folding potential. (**A**) The sequences selected for study by (**B**) ^1^H-NMR, (**C**) CD spectroscopy, and (**D**) thermal melting analysis (i.e., *T*_*m*_).

Lastly, we selected four PQSs found in the *Deinococcus-Thermus* phylum for biophysical characterization to establish whether they could adopt G4 folds; two sequences were selected from the order *Deinococcales* that occurred four times in the genome (DS2 and DS3) and two from the order *Thermales* that occurred four times in the genome (TS1 and TS4; Fig. 6A). After synthesis and purification of these DNA sequences, they were studied by ^1^H-NMR, CD spectroscopy, and *T*_*m*_ analysis. The two sequences studied in the *Deinococcales* order (Fig. 6A, DS2 and DS3) provided ^1^H-NMR peaks in the 10–12 ppm range indicative of G4 formation, on the basis of comparisons to the literature (Fig. 6B)^57^. These two sequences provided CD spectra supporting folding to parallel-stranded G4s, again on the basis of comparisons to the literature (Fig. 6C)^58,59^. In the final experiment, the thermal stabilities for these two sequences were measured and found to be >60 °C (Fig. 6D). These observations support the ability of two example PQSs from the *Deinococcales* order to fold to G4s under their normal growth temperatures (~37 °C); however, when the organism is thermally stressed these G4s will be much less stable.

When the two PQSs from the *Thermales* were inspected for G4 formation by the same biophysical methods, G4 folding was not observed (Fig. 6A–D). Inspection of the PQSs from the *Thermales* found they are longer and have larger loops than those from the *Deinococcales* (Fig. 6A). Because large loops are generally destabilizing to G4 folds^12^, the longer loops present in these sequences are the likely culprit for the failure of these sequences to fold. The biophysical interrogation of a few PQSs found in the *Deinococcus-Thermus* phylum suggests the possibility that some of these sequences can adopt G4 folds. Interestingly, both *D*. *radiodurans* and *T*. *aquaticus* are rich in helicases (13 and 19, respectively), but their roles are not fully understood. Because *T*. *aquaticus* has a larger fraction of its PQSs in coding regions, we speculate that some of these helicases assist processing of these G-rich sections of the genome. The data herein aid in determining that the PQSs from *Deinococcales* can fold under the living conditions of these bacteria (37–55 °C). As for the PQSs studied from *Thermales*, the two sequences studied did not fold; however, this initial, and limited study, does not represent the folding potential for all PQSs in these organisms.

## Conclusions

The present study inspected representative, sequenced genomes found in the NCBI database for PQSs. The initial analysis established the background density of PQSs (PQSs/Mbp) in many representative genomes across the kingdoms of life (Fig. 2). We found higher organisms with large genomes and 40 ± 10% GC content possess PQSs with densities ranging from 100–500 PQSs/Mbp. Additionally, the PQSs found were significantly enriched with >4 G tracks, a finding that supports a previous hypothesis of from our laboratory, in which additional G runs aid in maintaining G4 folding in the event that a critical G in G4 is oxidatively modified^16^. Next, this initial plot led to the observation that microorganisms are quite diverse in their densities of PQSs, and therefore, we focused our analysis on these genomes for parsing out PQSs. We found that in general PQS densities tracked with %GC content, in which high %GC genomes had greater numbers of PQSs (Fig. 3); however, a few phyla of microorganisms stood out as having possibly favorably evolved to contain PQSs, either on the whole genome scale or around regulatory regions. These phyla include the *Deinococcales-Thermales* (Fig. 4), the phytopathogens found in the *Xanthomonadales*, the *Cyanobacteria*, and the extremeophiles found in the kingdom of Archaea (Figs S5–S12). Collectively these observations suggest thermophilic microorganisms appear to favor PQSs in their genomes, with the exception of the *Xanthomonadales*.

Subsets of the genomes from the phyla of microorganisms having greater PQS densities than expected, and having annotated genomes, were inspected in greater detail. Two case studies are presented. For the radiation-resistant bacteria found in the order *Deinococcales*, the PQSs are enriched around TSSs on the template strand. Further, the PQSs in the *Deinococcales* were significantly enriched in >4 G tracks (Fig. 4D). In contrast, for the thermophiles found in the order *Thermales*, the PQSs are not enriched around TSSs or in any genic regions (Fig. 4). These two case studies suggest different evolutionary explanations for the functions PQSs may have in a genome.

On the one hand, radiation-resistant bacteria such as the *Deinococcales* maintain PQSs around TSSs that regulate transcription of genes. This observation suggests these PQSs may function in gene regulation and possibly aid in these types of cells to handle oxidative stress that occurs during radiation exposure^26,27^. Previous studies in microorganisms have found PQSs can regulate gene expression from a plasmid in *E*. *coli* cells^60^. In *D*. *radiodurans*, expression levels from PQS-bearing genes are downregulated with a G4-binding compound, and these compounds when administered to cells attenuate their ability to respond to stress^44,45^. Our laboratory has proposed in mammalian cells that G-rich promoter PQSs are sites of G oxidation for focusing DNA repair and regulating gene transcription^29,30,54,61^. Therefore, stress-resistant organisms such as *D*. *radiodurans* may have evolved to have PQSs around genes to function as antennas for reactive oxygen species to guide the cellular response to these radicals. Consistent with our conclusions, the addition of a G4-binding compound to *D*. *radiodurans* would interfere with the susceptibility of these G-rich sequences to be oxidized and block the DNA repair enzymes that drive gene transcription upon oxidation of promoter DNA sequences. Therefore, the G4-specific compounds may have blocked the natural cycle of G oxidation and G4 folding allowing the normal response to oxidative stress by *D*. *radiodurans*.

However, there remains a question that must be resolved: Why is there a strong bias for PQSs on the template strand flanking the TSS? In the few examples studied in mammalian cells (*VEGF*, *RAD17*, and *NTHL1*), PQSs in the template strand, when oxidatively modified near TSSs, downregulate transcription^29,30,62^, while oxidative modification of a PQS ~150 nts upstream of the TSS (*KRAS*) in the template strand can upregulate transcription^31^. The limited number of sequences studied cannot be harnessed to make strong claims; nonetheless, with the present data that is available, PQSs near the TSS in the template strand appear to downregulate transcription when oxidatively modified in mammalian cell culture. In the prokaryote *D*. *radiodurans*, the PQSs in promoters are favorably biased to the template strand around the TSS (Fig. 4C), and therefore, if our hypothesis holds in this organism, genes with promoter PQSs near the TSS should be downregulated. What is the benefit to downregulating many genes, especially those involved in oxidoreductase activity that includes enzymes for detoxification of reactive oxygen species? Future studies to address oxidation of the *D*. *radiodurans* genome and other model microorganisms and how this impacts gene expression profiles after oxidation will be critical for our understanding of the regulatory function of promoter PQSs in these organisms. We have developed a method for sequencing the G oxidation product OG on the mammalian genome scale^63^ and have the ability to site-specifically modify promoters in a reporter gene found in a plasmid^29^, providing us with the tools to address this question and others in the near future.

On the other hand, thermophilic organisms possess greater densities of PQSs because their genomes are GC rich as consequence of needing to increase the overall stability of duplex to survive at high temperatures. Although, why PQSs have evolved at a greater frequency than expected by random chance is mysterious (Figs 2 and 3). One possibility is that evolution toward runs of G in duplex DNA leads to greater thermal stability than having the same number of Gs randomly distributed. This claim is consistent with *T*_*m*_ studies on model duplexes, in which G runs are more thermally stable than when they have intervening non-G nucleotides^64^. Therefore, selection of G runs while having a high GC% in the genomes unavoidably yields greater densities of PQSs. These fascinating observations regarding genome PQSs in microorganisms will provide the background for many more studies regarding these G-rich and oxidation-sensitive sequences in the future.

## Electronic supplementary material

Supplementary Information
