# Supplementary material for: Case studies on potential G-quadruplex-forming sequences from the bacterial orders Deinococcales and Thermales derived from a survey of published genomes
Source: Sci Rep. 2018 Oct 24;8:15679. doi: 10.1038/s41598-018-33944-4 (PMC6200779; doi:10.1038/s41598-018-33944-4)
Supplement: Supplementary file 1 — Supplementary Information [file 41598_2018_33944_MOESM1_ESM.pdf]

Supporting Information

Case studies on potential G-quadruplex forming sequences from the bacterial orders *Deinococcales* and *Thermales* derived from a survey of published genomes

Yun Ding*, Aaron M. Fleming, and Cynthia J. Burrows*
Department of Chemistry, University of Utah, 315 South 1400 East, Salt Lake City, UT, 84112-0850, United States

Item	Page
**Complete biophysical characterization methods**	S2
**Figure S1**. Frequency of >4 G tracks in randomized genomes of known GC%.	S4
**Figure S2**. Additional PQS densities in the phyla found in the NCBI database.	S5
**Figure S3.** PQS distributions in the orders of Archaea.	S6
**Figure S4.** PQS densities in the phylum of *Cyanobacteria*.	S7
**Figure S5.** PQS distributions around the TSSs in the *Alphaproteobacteria*.	S8
**Figure S6.** PQS distributions around the TSSs in the *Betaproteobacteria*.	S9
**Figure S7.** PQS distributions around the TSSs in the *Gammaproteobacteria.*	S10
**Figure S8.** PQS distributions around the TSSs in the *Delta/epsilon proteobacteria.*	S11
**Figure S9.** PQS distributions around the TSSs in the *Actinobacteria.*	S12
**Figure S10.** PQS distributions around the TSSs in the *Chloroflexi.*	S13
**Figure S11.** PQS distributions around the TSSs in the *Cyanobacteria.*	S14
**Figure S12.** PQS distributions around the TSSs in the *Firmicutes.*	S15
**Figure S13.** PCA analysis of the PQS distribution around the TSSs in the phylum of *Deinococcus-Thermus.*	S16
**Table S1.** Genes in *Deinococcus radiodurans* with a PQS around 100 nt of the TSS on the template strand involved in oxidoreductase activity from the PANTHER classification system.	S17
**Table S2.** Genes in *Xanthomonas campestris* with a PQS around 100 bp of the TSS on the template strand involved in oxidoreductase activity from the PANTHER classification system.	S18
**Table S3.** Genes in *Xanthomonas campestris* with a PQS around 100 bp of the TSS involved in the GO term response to stress.	S19

Complete biophysical characterization methods

Oligomer preparation. The oligomers were synthesized by the DNA/Peptide core facility at the University of Utah using commercially available phosphoramidites and a standard solid-phase synthesis protocol. The crude oligomers were purified using a semi-preparative, anion-exchange HPLC column running line A = 1:9 ddH_2O:MeCN, and line B = 20 mM LiOAc (pH 7) with 1 M LiCl in 1:9 ddH_2O:MeCN and a flow rate = 3 mL/min while monitoring the elution via the absorbance at 260 nm. After purification, the oligomers were dialyzed against ddH_2O for 36 h while changing the water three times to remove the purification salts. The dialyzed samples were lyophilized to dryness and resuspended in ddH_2O. The concentrations were determined by the absorbance at 260 nm using the primary sequence to estimate the extinction coefficients. All oligomers were stored at -20 °C when not being studied. The G4 strands were annealed in the desired salt and buffer by heating them to 90 °C for 5 min and then slowly cooling the samples to room temperature over ~4 h. After reaching room temperature, the samples were stored at 4 °C for at least 24 h prior to their study.

^{1}H-NMR Analysis. The PQS samples were annealed in 300 µL at a 300 µM concentration in 20 mM KP_i (pH 7.0) and 50 mM KCl in 9:1 H_2O:D_2O. The annealed samples were placed in a D_2O-matched Shigemi NMR tube. The samples were analyzed on an 800-MHz NMR spectrometer (Varian, Inc.) with the temperature set to 24 °C. Each sample was scanned 2,048 times using the Watergate solvent suppression pulse sequence. The data were analyzed and plotted using the instrument's software.

Circular Dichroism Analysis. The PQS samples were annealed at 10 μM concentration in 20 mM lithium cacodylate buffer (pH 7.4) with 140 mM KCl and 12 mM NaCl. The samples were placed in a 0.2-cm quartz cuvette for circular dichroism (CD) analysis at 20 °C (Jasco J-815 circular dichroism spectrometer). The recorded data were solvent background subtracted and then normalized on the y-axis to units of molar ellipticity ([Θ]) for plotting and comparative purposes.

Thermal Melting Analysis. The thermal melting (T_m) values were determined on samples of 5 μM oligomer in buffered solutions with human physiological K^+ and Na^+ concentrations (20 mM lithium cacodylate pH 7.4, 140 mM KCl, and 12 mM NaCl). The melting experiments were initiated by thermally equilibrating the samples at 20 °C for 10 min followed by heating at 0.5 °C/min and equilibrating at each 1 °C increment for 1 min. Readings at 260 and 295 nm were taken after each 1 °C change in the temperature starting at 20 °C up to 95 °C. Plots of absorbance at 295 nm vs. temperature were constructed, and the T_m values were determined by a two-point analysis protocol using the instrument's software (Shimadzu Scientific UV-1800 spectrometer).

Figure S1. Frequency of >4 G tracks in randomized genomes of known GC%.

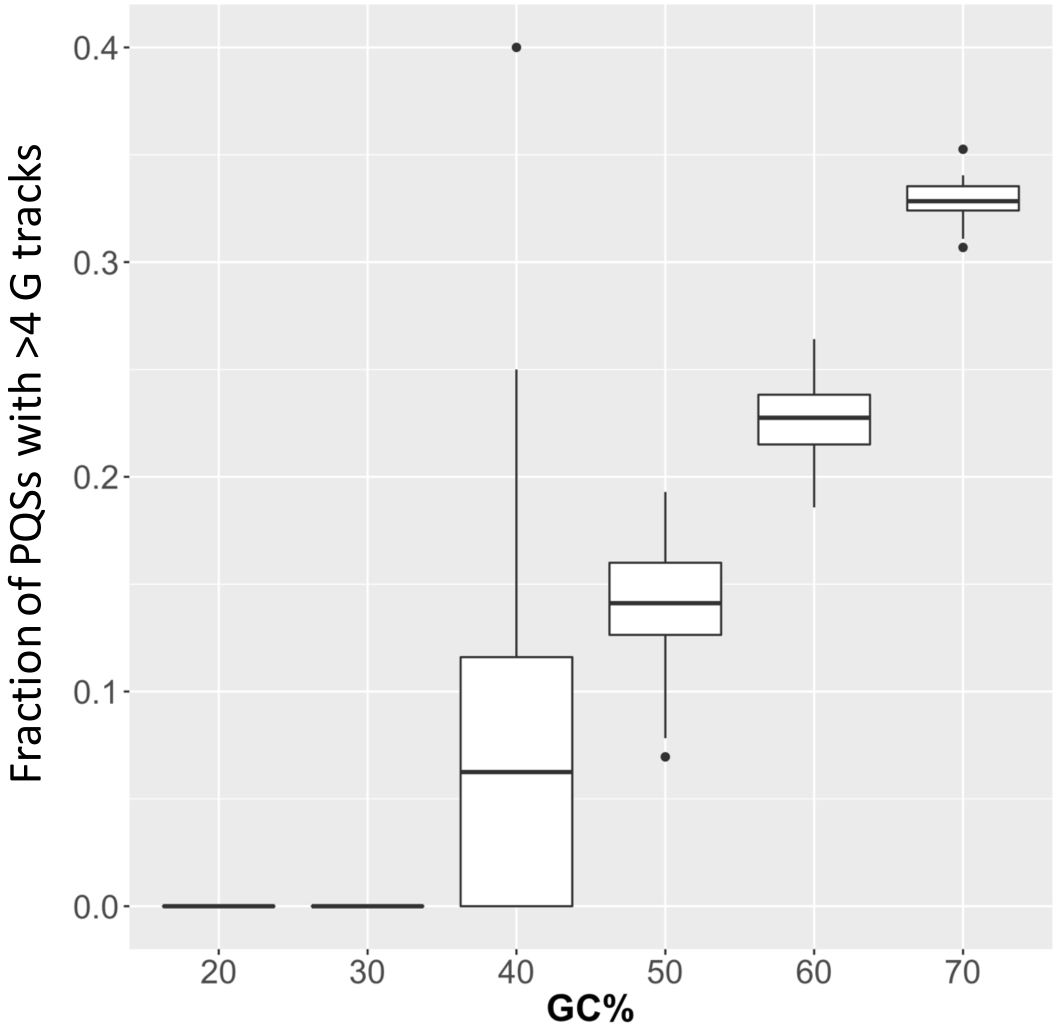

A collection of 50 randomized genomes of 2 million base pairs each were generated at each specific GC%. The fraction of PQSs with > 4 G tracks was calculated for each genome and the results were plotted in the boxplot shown above.

Figure S2. Additional PQS densities in the phyla found in the NCBI database.

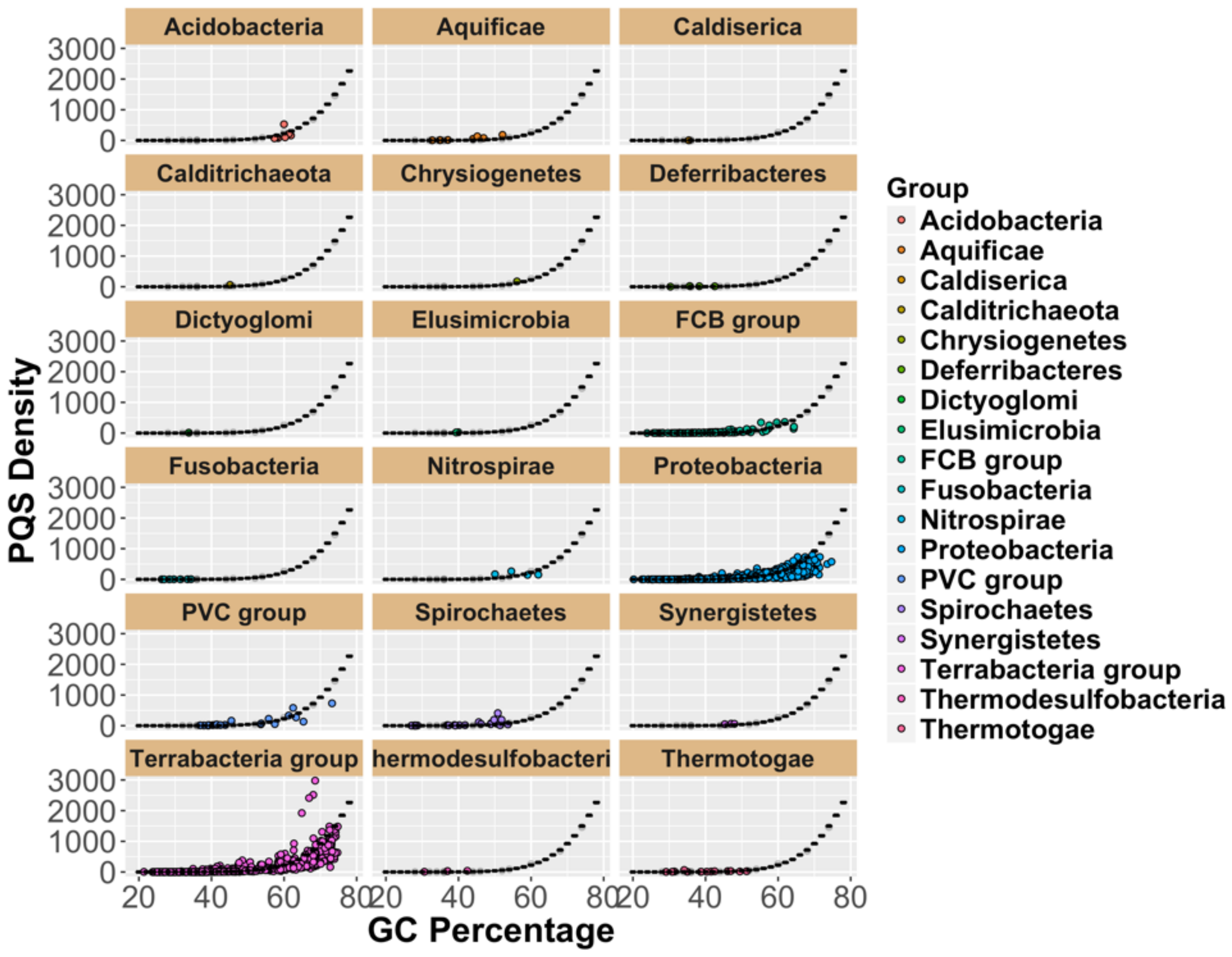

The black lines represent the PQS densities from the randomized genomes analyzed with known GC% content.

Figure S3. PQS distributions in the orders of Archaea.

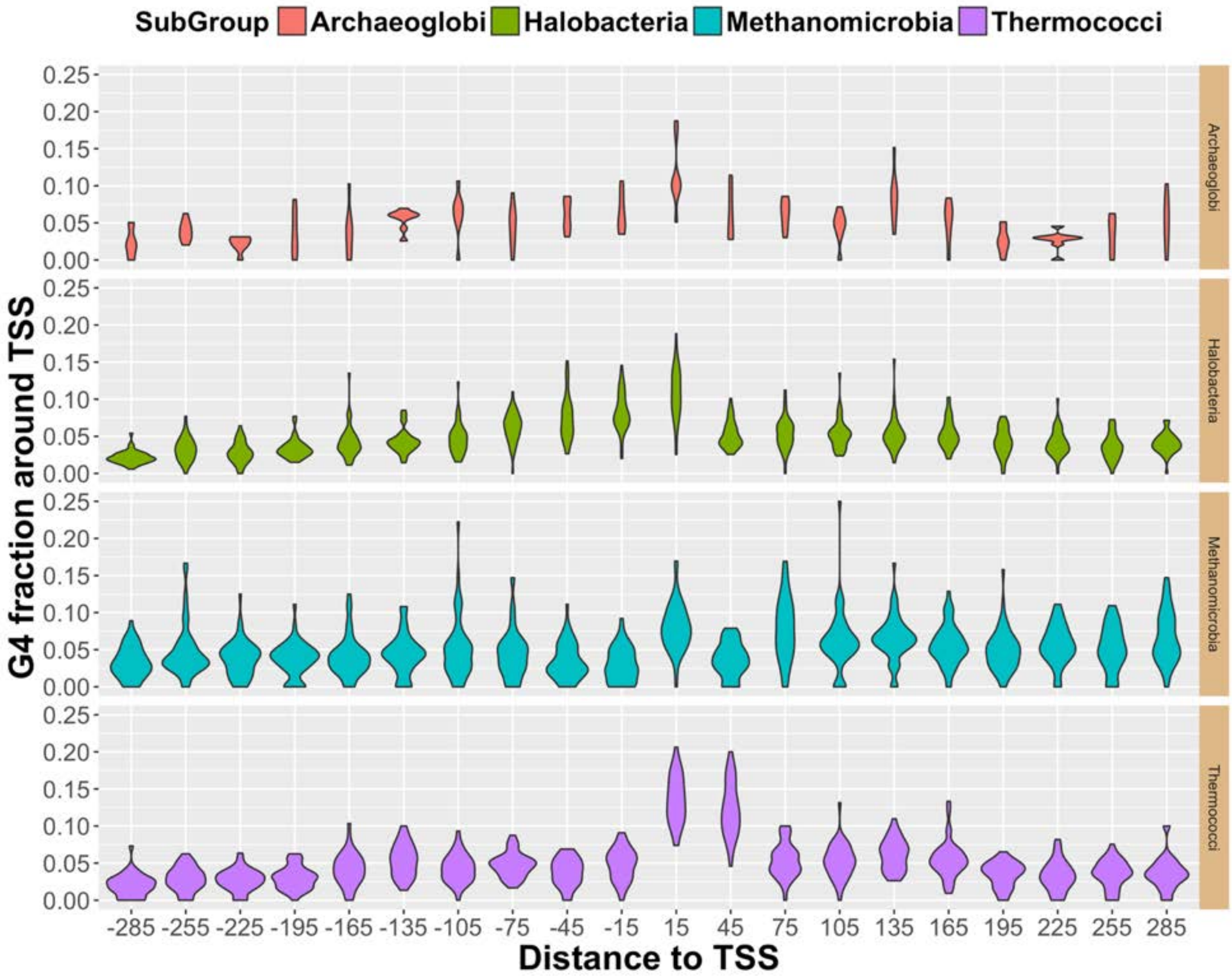

Figure S4. PQS densities in the phylum of *Cyanobacteria*.

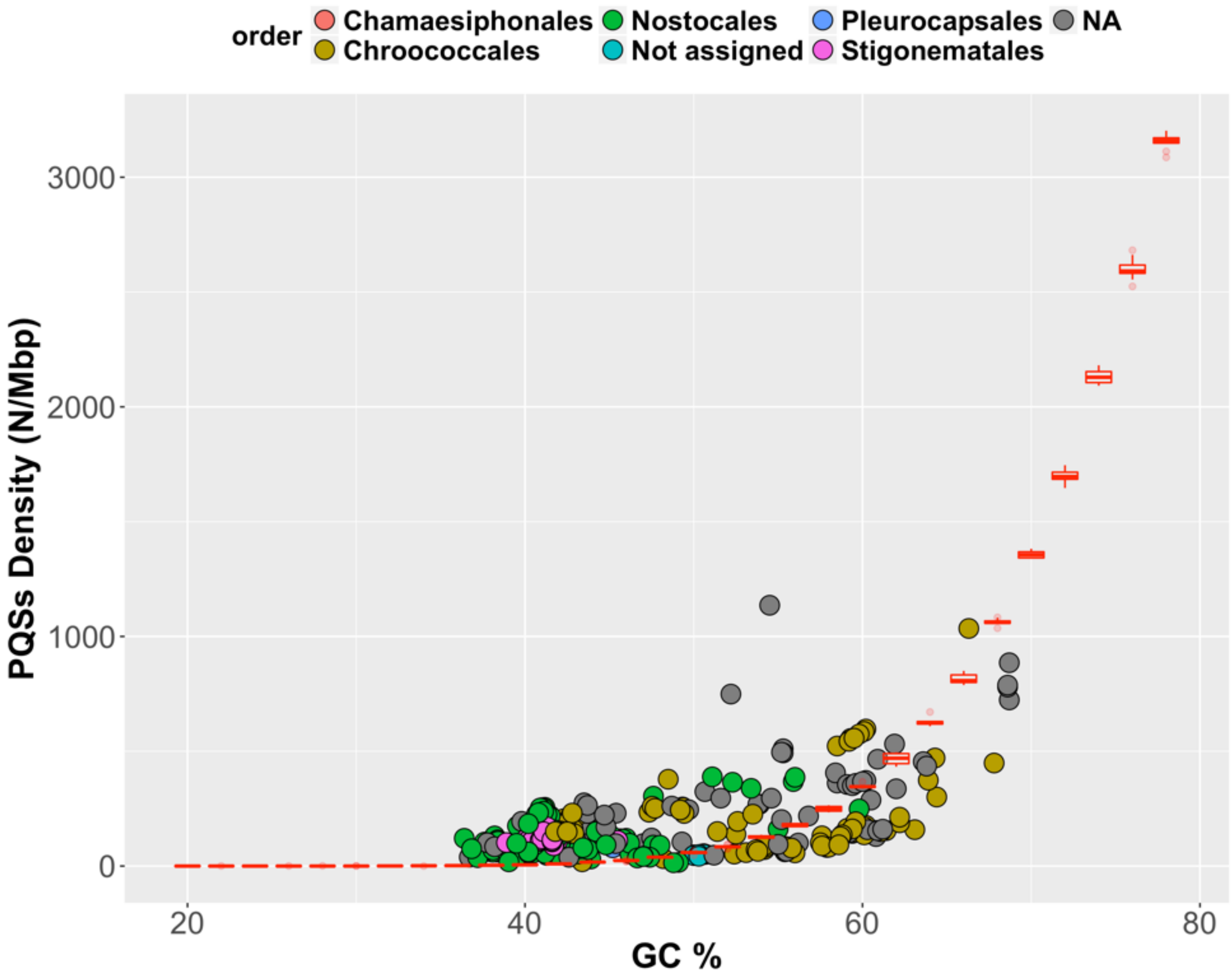

Figure S5. PQS distributions around the TSSs in the *Alphaproteobacteria*.

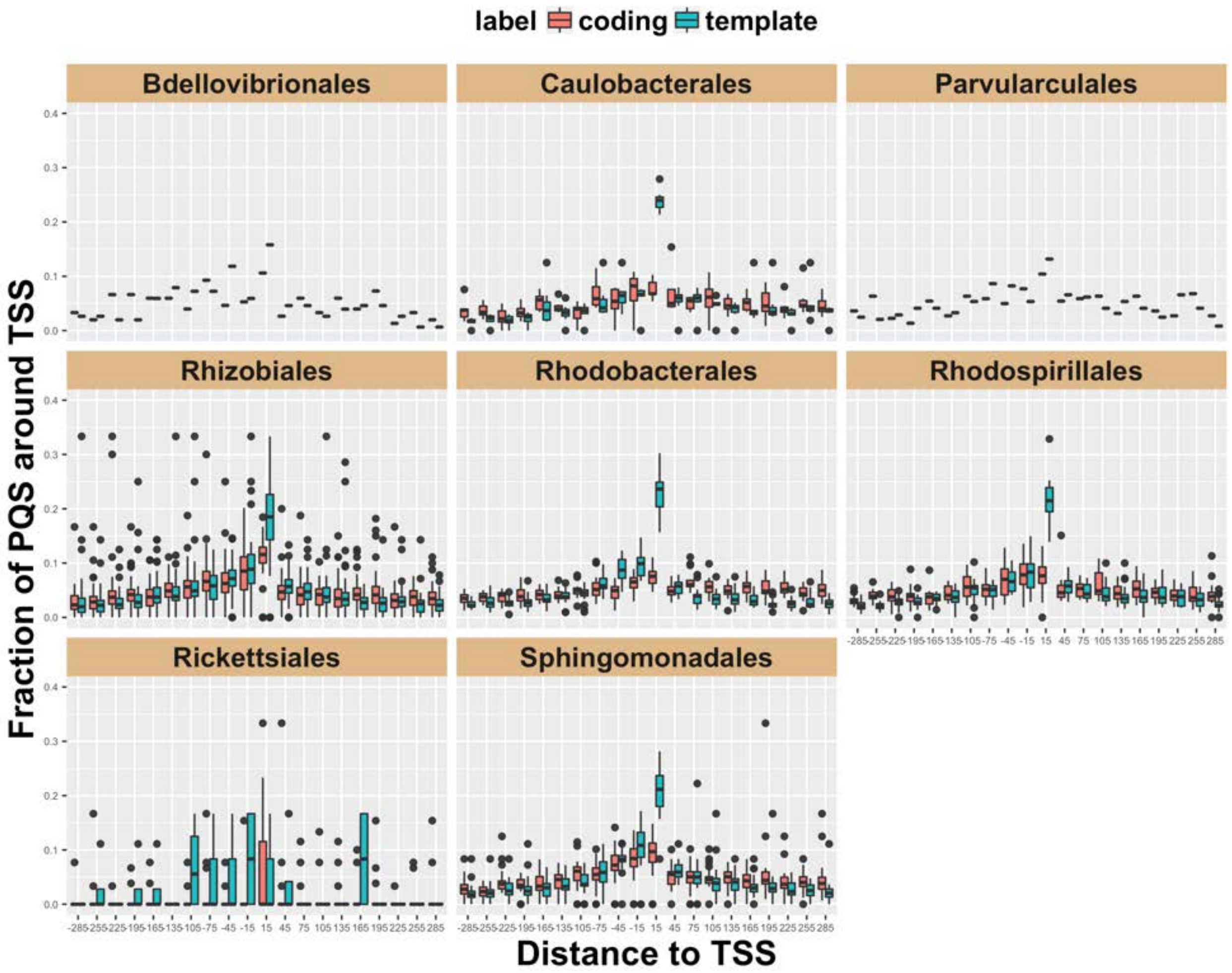

Figure S6. PQS distributions around the TSSs in the *Betaproteobacteria*.

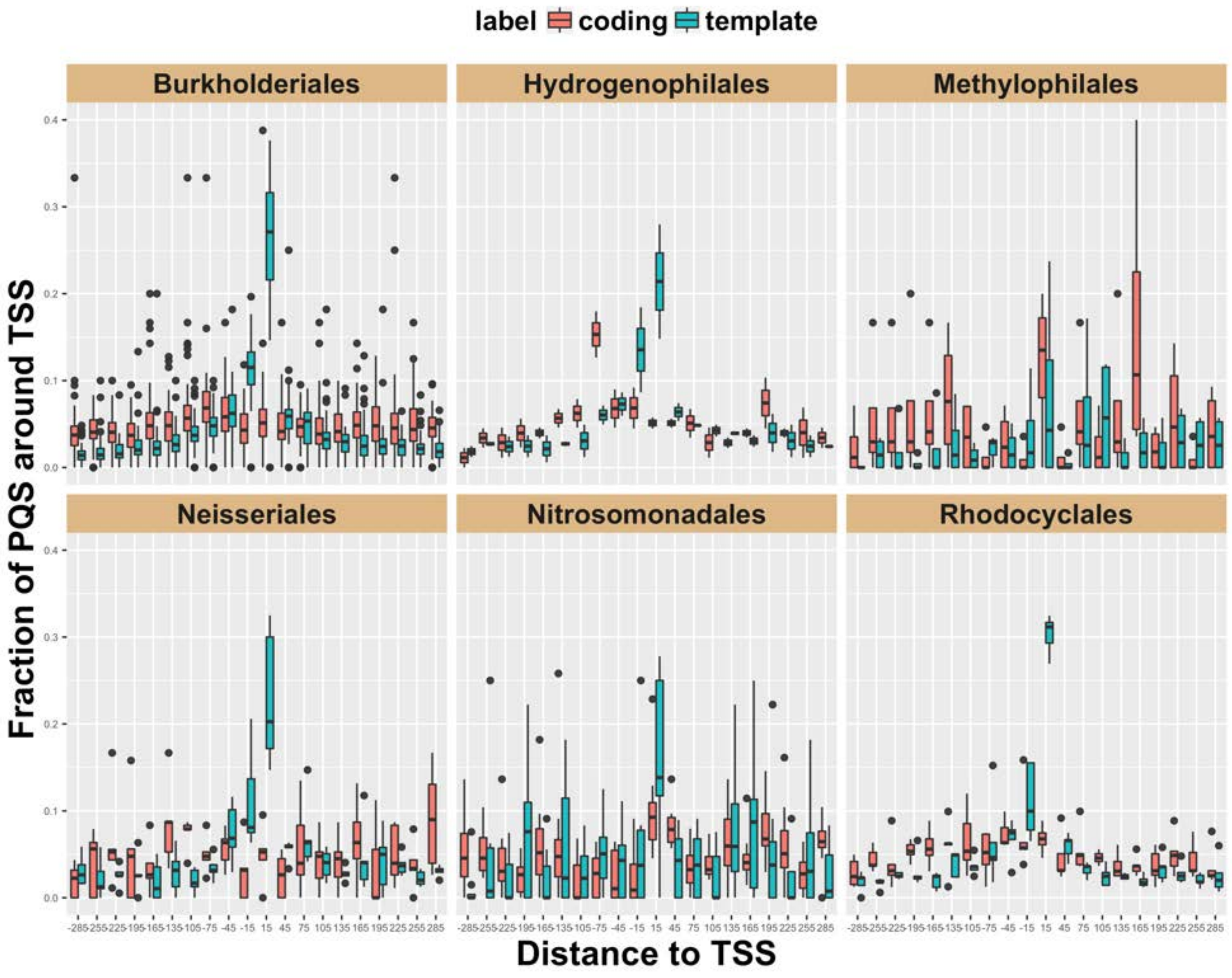

Figure S7. PQS distributions around the TSSs in the *Gammaproteobacteria.*

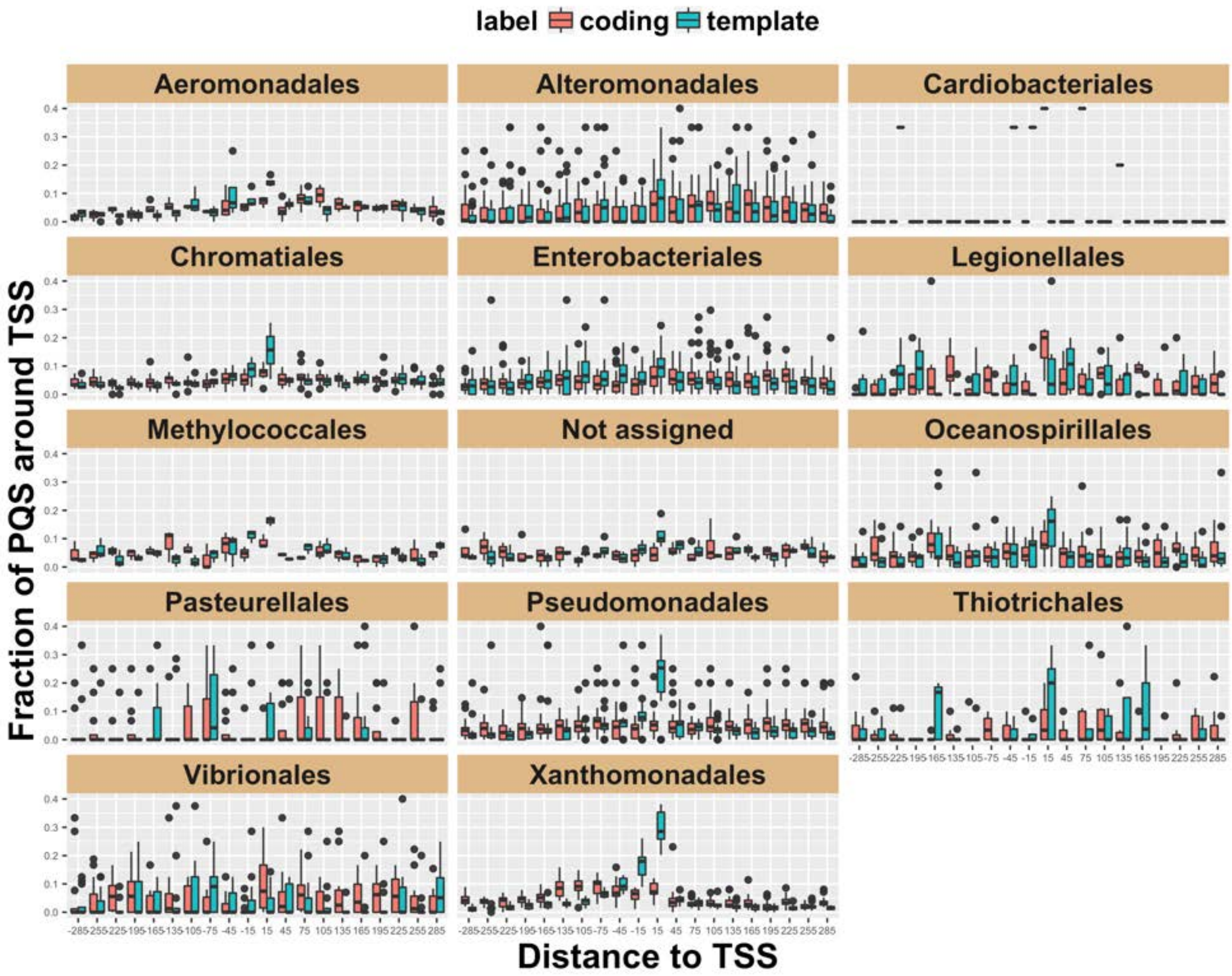

Figure S8. PQS distributions around the TSSs in the *Delta/epsilon proteobacteria.*

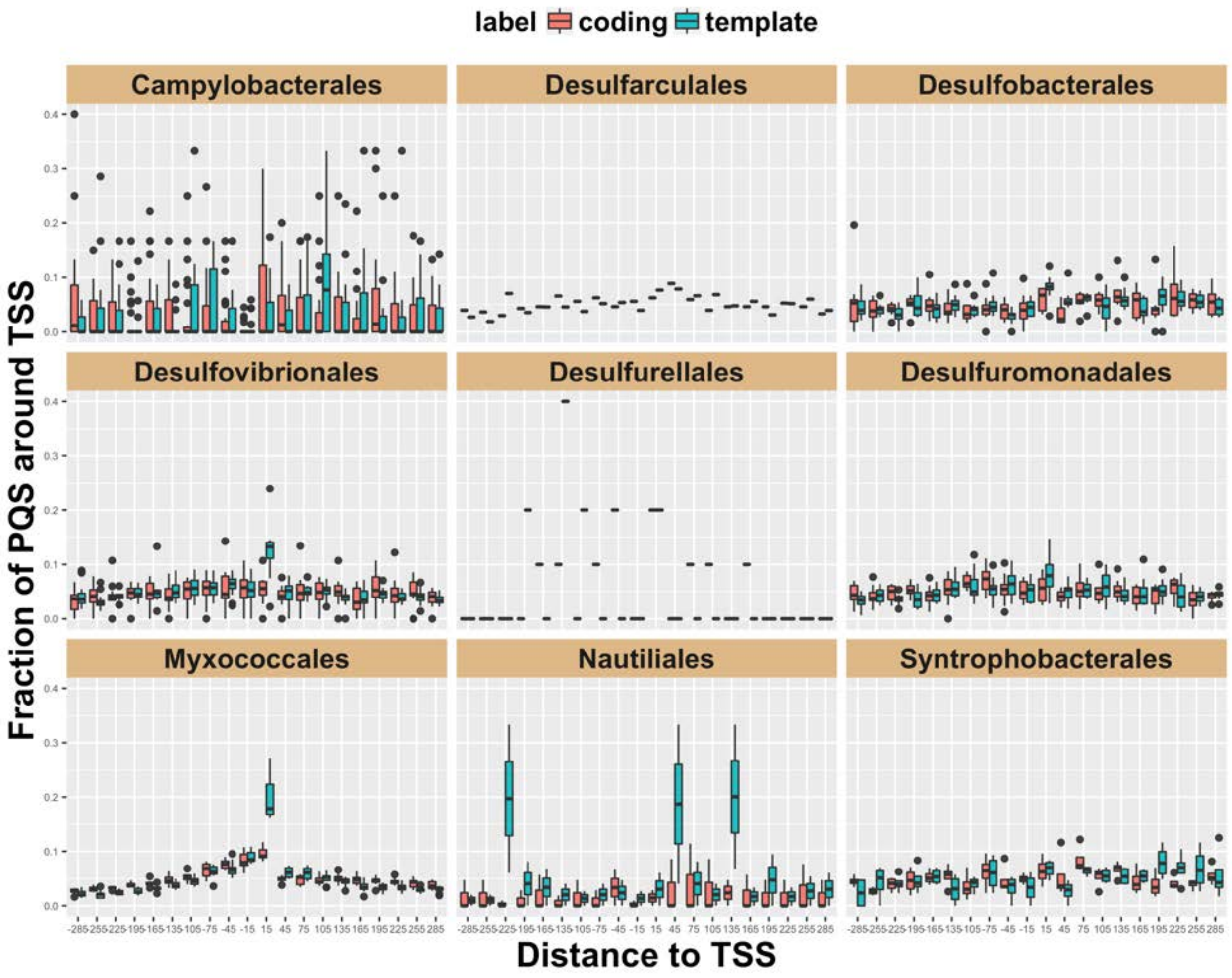

Figure S9. PQS distributions around the TSSs in the *Actinobacteria.*

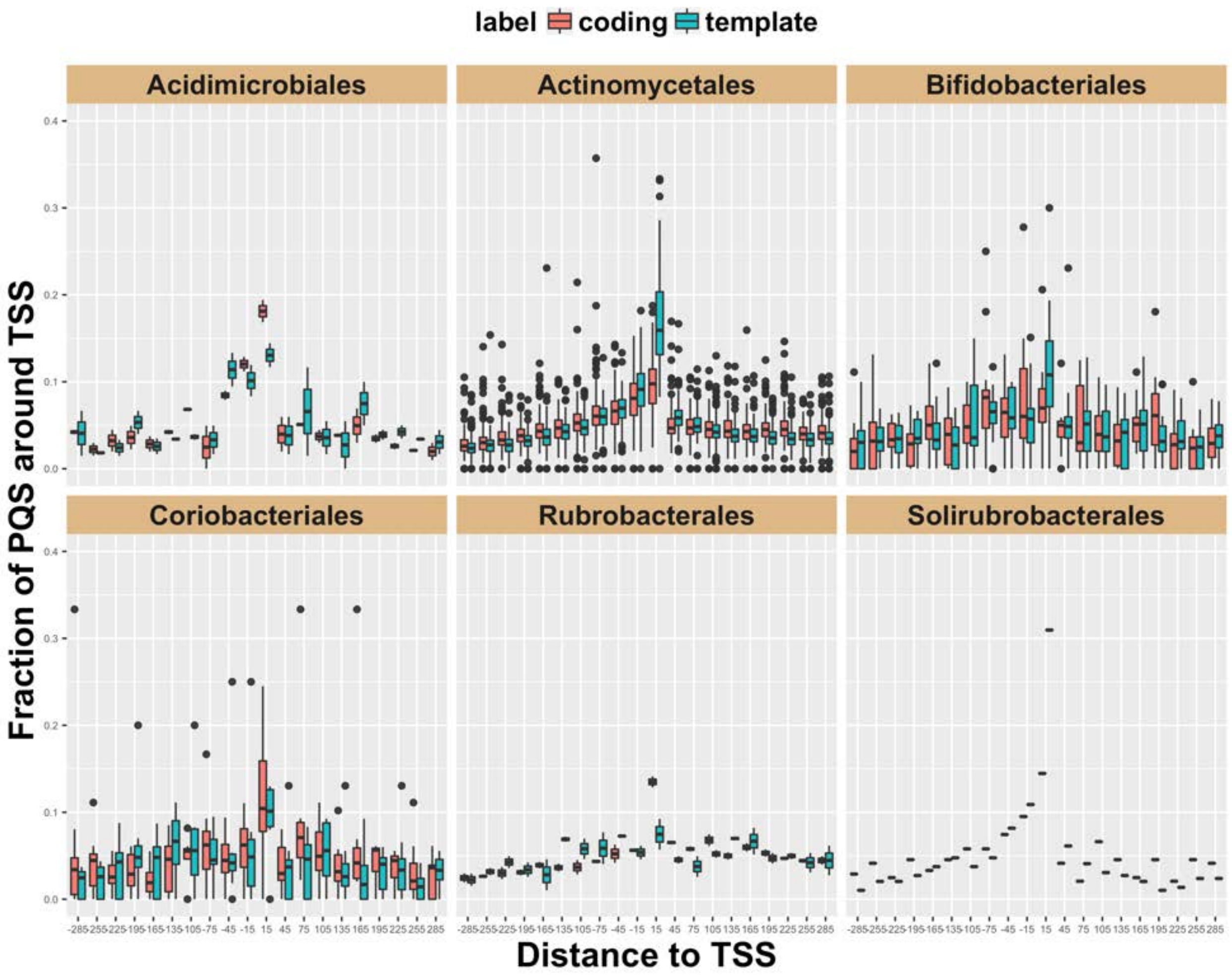

Figure S10. PQS distributions around the TSSs in the *Chloroflexi.*

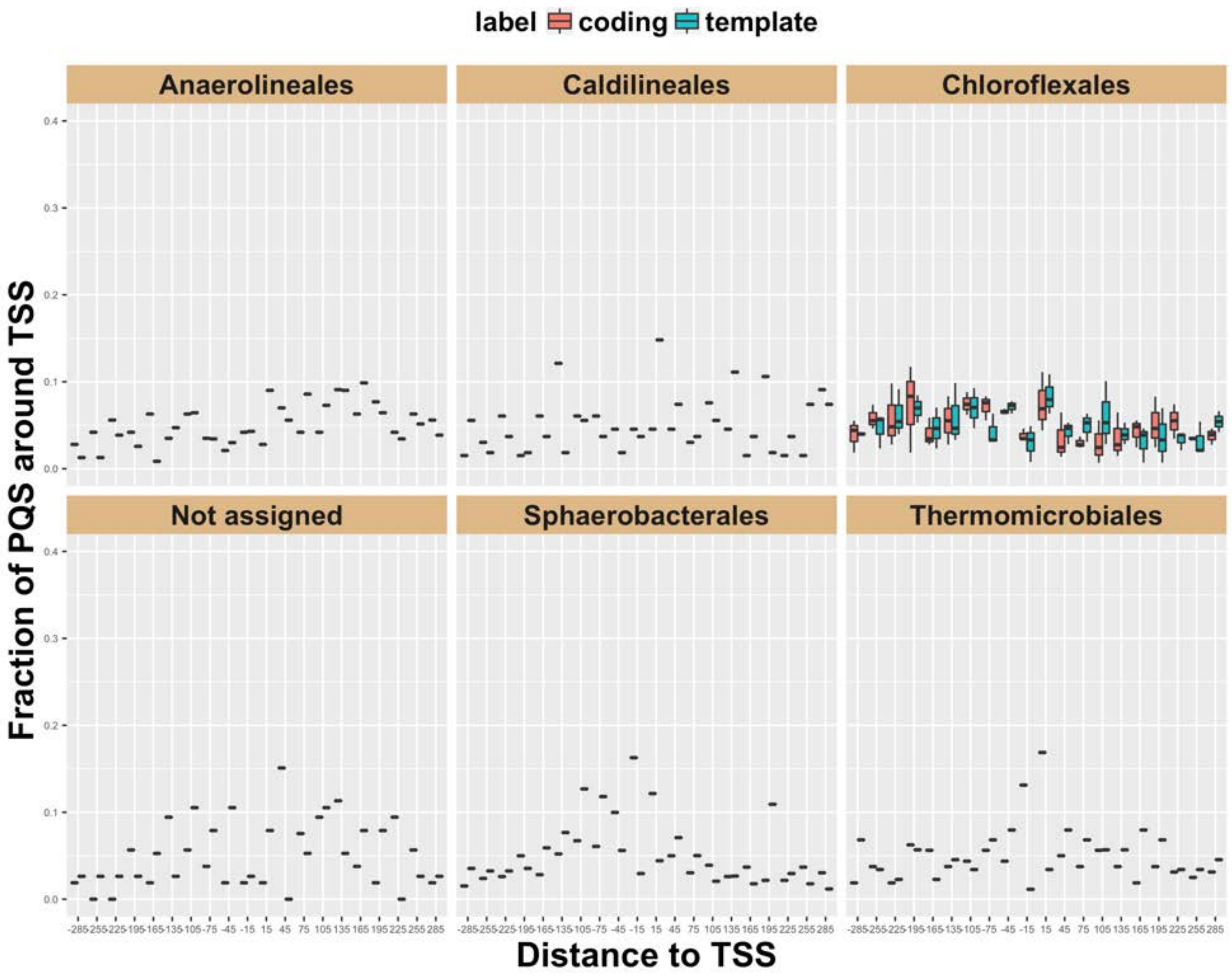

Figure S11. PQS distributions around the TSSs in the *Cyanobacteria.*

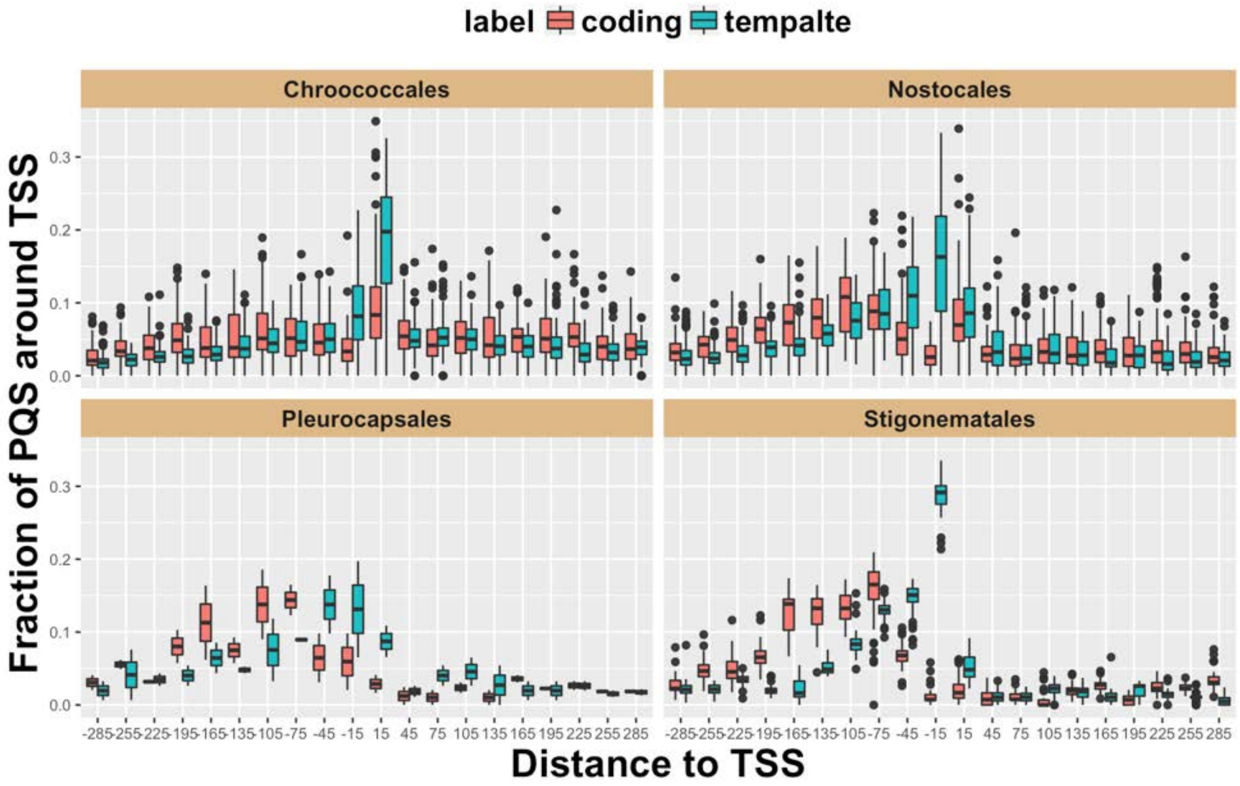

Figure S12. PQS distributions around the TSSs in the *Firmicutes.*

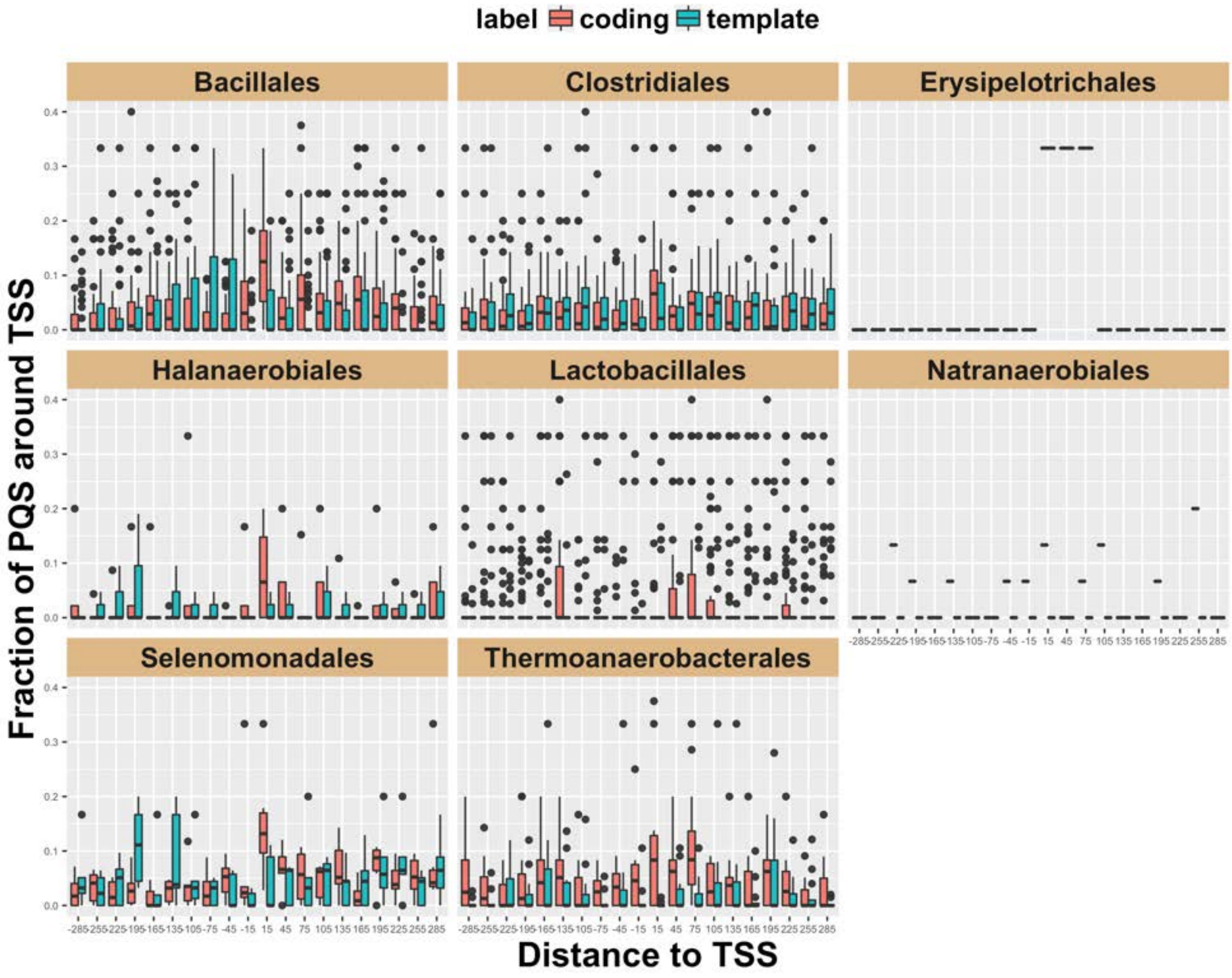

Figure S13. PCA analysis of the PQS distribution around the TSSs in the phylum of *Deinococcus-Thermus.*

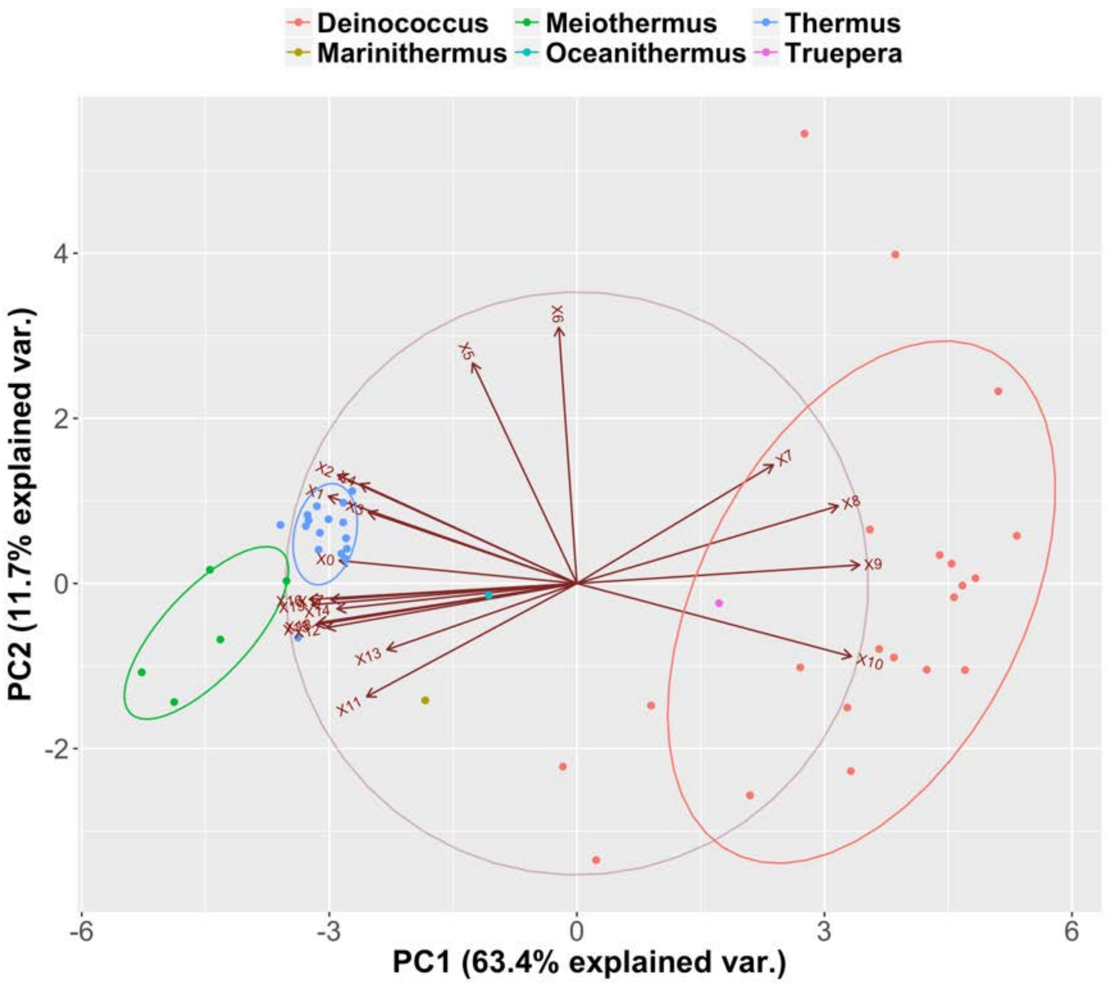

Data are color coded by genera. The genera of *Deinococcus* and *Truepera* belong to the order of *Deinococcales*, and the rest of genera belong to the order of *Thermales*. Both orders are well separated in this PCA plot.

Table S1. Genes in *Deinococcus radiodurans* with a PQS around 100 nt of the TSS on the template strand involved in oxidoreductase activity from the PANTHER classification system.

DR_1769	Desiccation/radiation resistance protein DR_1769;DR_1769;ortholog
DR_0073	Phosphate acetyltransferase;pta;ortholog
DR_1505	NADH-quinone oxidoreductase subunit B;nuoB;ortholog
DR_1698	tRNA-dihydrouridine(20/20a) synthase;DR_1698;ortholog
DR_1399	Oxidoreductase, putative;DR_1399;ortholog
DR_A0343	Succinate-semialdehyde dehydrogenase [NADP(+)];ssdA;ortholog
DR_1291	D-3-phosphoglycerate dehydrogenase;DR_1291;ortholog
DR_2140	Histidinol dehydrogenase;hisD;ortholog
DR_A0164	Thioredoxin 1;DR_A0164;ortholog
DR_1208	Bacterioferritin comigratory protein;DR_1208;ortholog
DR_0833	Histone deacetylase/AcuC/AphA family protein;DR_0833;ortholog
DR_1980	Uncharacterized protein;DR_1980;ortholog
DR_1968	Nitroreductase;DR_1968;ortholog
DR_A0339	Tryptophan 2,3-dioxygenase;kynA;ortholog
DR_0861	Phytoene dehydrogenase;DR_0861;ortholog
DR_1724	Uncharacterized protein;DR_1724;ortholog
DR_A0243	Flavohemoprotein;hmp;ortholog
DR_A0276	Malate oxidoreductase;DR_A0276;ortholog
DR_2261	Aldo/keto reductase;DR_2261;ortholog
DR_1638	Uncharacterized protein;DR_1638;ortholog
DR_A0126	Aldehyde dehydrogenase;DR_A0126;ortholog
DR_1026	Oxidoreductase;DR_1026;ortholog
DR_A0178	Xanthine dehydrogenase, C-terminal subunit;DR_A0178;ortholog
DR_0733	Histidine biosynthesis bifunctional protein HisIE;hisI;ortholog
DR_0184	Enoyl-CoA hydratase, putative;DR_0184;ortholog
DR_0571	Uncharacterized protein;DR_0571;ortholog
DR_1731	Glycolate oxidase subunit GlcD;DR_1731;ortholog
DR_0336	Fatty-acid--CoA ligase, putative;DR_0336;ortholog
DR_0496	Ferredoxin/ferredoxin--NADP reductase, putative;DR_0496;ortholog

Table S2. Genes in *Xanthomonas campestris* with a PQS around 100 bp of the TSS on the template strand involved in oxidoreductase activity from the PANTHER classification system.

XCC0934	Zinc-type alcohol dehydrogenase-like protein;XCC0934;ortholog
XCC3355	Acetoacetyl-coA reductase;phbB;ortholog
XCC0833	Alkyl hydroperoxide reductase subunit F;ahpF;ortholog
XCC0432	Tryptophan 2,3-dioxygenase;kynA;ortholog
XCC0766	Voltage-gated potassium channel beta subunit;XCC0766;ortholog
XCC0029	Alcohol dehydrogenase;yahK;ortholog
XCC3669	Oxidoreductase;XCC3669;ortholog
XCC3439	6-phosphogluconate dehydrogenase;gndA;ortholog
XCC3864	Oxidoreductase;XCC3864;ortholog
XCC3173	Sulfite reductase [NADPH] flavoprotein alpha-component;cysJ;ortholog
XCC0037	Pirin;XCC0037;ortholog
XCC3695	Oxidoreductase;XCC3695;ortholog
XCC3835	Bifunctional protein PutA;putA;ortholog
XCC3651	Peptide methionine sulfoxide reductase MsrB;msrB;ortholog
XCC0438	Homogentisate 1,2-dioxygenase;hmgA;ortholog
XCC3949	Catalase;catB;ortholog
XCC1079	Oxidoreductase;XCC1079;ortholog
XCC2225	C-type cytochrome biogenesis protein/thioredoxin dsbE or;dsbE;ortholog
XCC3181	Siroheme synthase;cysG;ortholog
XCC2238	Gamma-glutamyl phosphate reductase;proA;ortholog
XCC1503	UDP-glucose dehydrogenase;ugd;ortholog
XCC1414	Ferredoxin-NADP reductase;fpr;ortholog
XCC4107	Mannitol dehydrogenase;mtlD;ortholog
XCC0191	Superoxide dismutase [Cu-Zn];sodC2;ortholog
XCC2185	Bifunctional protein FolD;folD;ortholog
XCC4065	Oxidoreductase;XCC4065;ortholog
XCC3691	Cyanide insensitive terminal oxidase;cioA;ortholog
XCC0031	Glutamate synthase, beta subunit;gltD;ortholog
XCC3986	Ribonucleoside-diphosphate reductase;nrdA;ortholog
XCC2554	Uncharacterized protein;XCC2554;ortholog
XCC1112	Glycine dehydrogenase (decarboxylating);gcvP;ortholog
XCC1485	Dihydrolipoyl dehydrogenase;ldp;ortholog
XCC0945	L-threonine 3-dehydrogenase;tdh;ortholog

Table S3. Genes in *Xanthomonas campestris* with a PQS around 100 bp of the TSS involved in the GO term response to stress.

XCC3026	Crossover junction endodeoxyribonuclease RuvC;ruvC;ortholog
XCC1359	Ribonuclease HII;rnhB;ortholog
XCC3651	Peptide methionine sulfoxide reductase MsrB;msrB;ortholog
XCC2268	Phosphoglycolate phosphatase;gph;ortholog
XCC3949	Catalase;catB;ortholog
XCC0705	Two-component system sensor protein;kdpD;ortholog
XCC2418	A/G-specific adenine glycosylase;mutY;ortholog
XCC0187	Ammonium transporter;amtB;ortholog
XCC2773	PKHD-type hydroxylase XCC2773;XCC2773;ortholog
XCC1521	Ribonuclease T;rnt;ortholog
XCC3035	Alpha,alpha-trehalose-phosphate synthase;XCC3035;ortholog
XCC1047	Low molecular weight heat shock protein;hspA;ortholog
XCC0191	Superoxide dismutase [Cu-Zn];sodC2;ortholog
